# An Engineered N-Glycosylated Dengue Envelope Protein Domain III Facilitates Epitope-Directed Selection of Potently Neutralizing and Minimally Enhancing Antibodies

**DOI:** 10.1021/acsinfecdis.4c00058

**Published:** 2024-06-29

**Authors:** Napon Nilchan, Romchat Kraivong, Prasit Luangaram, Anunyaporn Phungsom, Mongkhonphan Tantiwatcharakunthon, Somchoke Traewachiwiphak, Tanapan Prommool, Nuntaya Punyadee, Panisadee Avirutnan, Thaneeya Duangchinda, Prida Malasit, Chunya Puttikhunt

**Affiliations:** † Molecular Biology of Dengue and Flaviviruses Research Team, Medical Molecular Biotechnology Research Group National Science and Technology Development Agency (NSTDA), Pathum Thani 12120, Thailand; ‡ Medical Biotechnology Research Unit, National Center for Genetic Engineering and Biotechnology (BIOTEC), National Science and Technology Development Agency (NSTDA), Pathum Thani 12120, Thailand; § Siriraj Center of Research Excellence in Dengue and Emerging Pathogens Mahidol University, Bangkok 10700, Thailand; ∥ Division of Dengue Hemorrhagic Fever Research, Faculty of Medicine Siriraj Hospital, Mahidol University, Bangkok 10700, Thailand

**Keywords:** dengue, envelope, domain III, glycan, shield, epitope

## Abstract

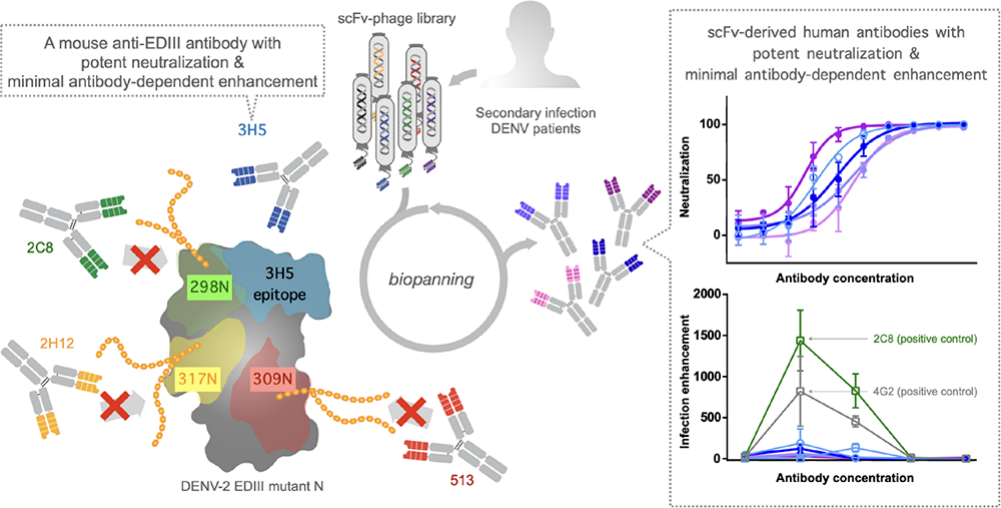

The envelope protein of dengue virus (DENV) is a primary target of the humoral immune response. The domain III of the DENV envelope protein (EDIII) is known to be the target of multiple potently neutralizing antibodies. One such antibody is 3H5, a mouse antibody that binds strongly to EDIII and potently neutralizes DENV serotype 2 (DENV-2) with unusually minimal antibody-dependent enhancement (ADE). To selectively display the binding epitope of 3H5, we strategically modified DENV-2 EDIII by shielding other known epitopes with engineered N-glycosylation sites. The modifications resulted in a glycosylated EDIII antigen termed “EDIII mutant N”. This antigen was successfully used to sift through a dengue-immune scFv-phage library to select for scFv antibodies that bind to or closely surround the 3H5 epitope. The selected scFv antibodies were expressed as full-length human antibodies and showed potent neutralization activity to DENV-2 with low or negligible ADE resembling 3H5. These findings not only demonstrate the capability of the N-glycosylated EDIII mutant N as a tool to drive an epitope-directed antibody selection campaign but also highlight its potential as a dengue immunogen. This glycosylated antigen shows promise in focusing the antibody response toward a potently neutralizing epitope while reducing the risk of antibody-dependent enhancement.

## Introduction

The humoral response to dengue virus (DENV) infection primarily targets the envelope (E) protein.^1,2^ While anti-E antibodies can offer protection and neutralize the virus, many of these antibodies can cause antibody-dependent enhancement (ADE). This phenomenon occurs when subneutralizing level of antibodies or non-neutralizing antibodies enhance viral infection in immune cells bearing Fc gamma receptors.^2−5^ ADE is associated with severe forms of dengue infection, presenting a major hurdle in DENV vaccine development.^6−8^

The properties and functions of anti-E antibodies are closely linked to their binding epitopes. For instance, antibodies that target the fusion loop epitope (FLE) generally exhibit low to moderate neutralization with prominent ADE activities, while those that target the domain III of the envelope (EDIII) typically exhibit robust neutralization capability.^5,9,10^ Even though anti-EDIII constitute a small fraction and contribute minimally to the overall serum neutralization,^11^ monoclonal anti-EDIII are some of the most potent neutralizing antibodies and are often associated with minimal or absent of ADE.^12−15^ Moreover, DENV EDIII is the target of type-specific (TS) antibodies that have been correlated with a long-lasting immunity against homotypic infection.^16−18^ Consequently, DENV EDIII stands out as an attractive subunit vaccine candidate.

**Figure 1:**
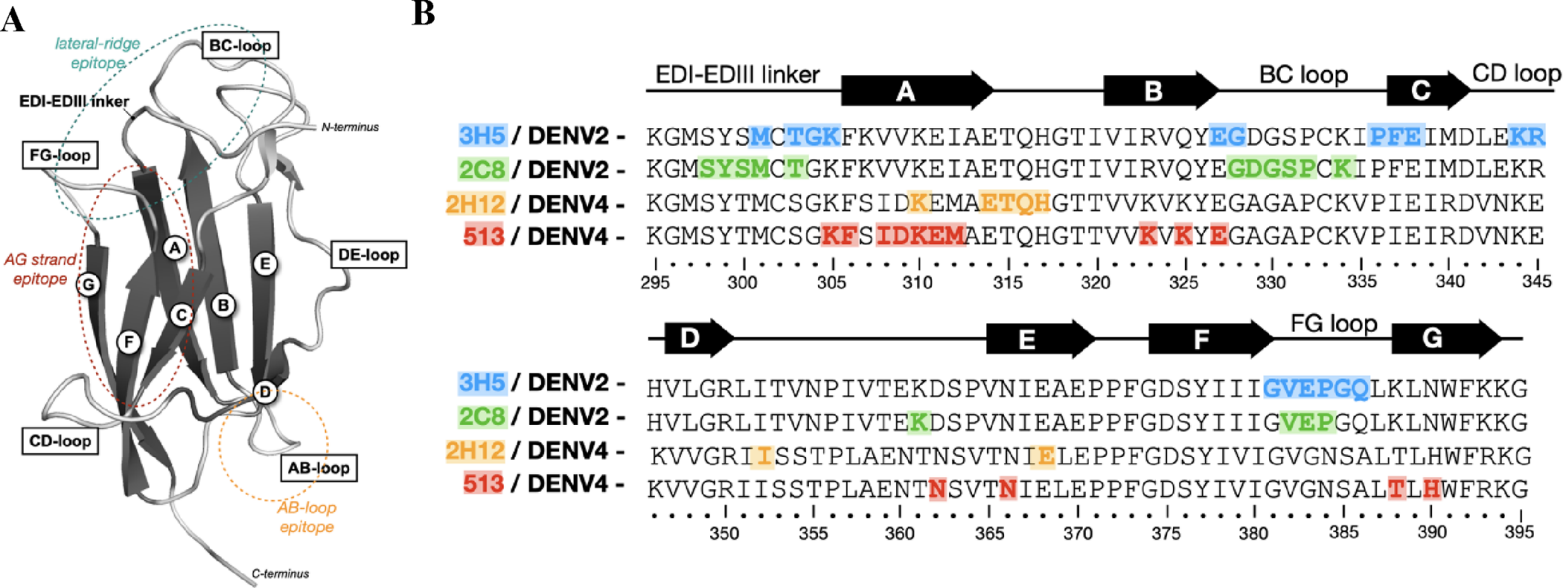
Structure and epitopes of DENV EDIII. (A) Structure of DENV-2 EDIII with three epitopes, AG-strand, AB-loop, and lateral-ridge epitopes circled in dotted line. The structure was extracted from PDB 1OAN (amino acid residues 298–397). (B) Epitopes of anti-EDIII antibodies used in this study. The epitope residues are highlighted in colored corresponding to each antibody.

Structurally, DENV EDIII is a self-contained 100-amino acid-long immunoglobulin-like domain that can be expressed independently of other domains. The EDIII harbors three main epitope regions—an AB loop, an AG strand, and a lateral ridge (Figure 1A). The AB loop epitope is largely conserved across DENV serotypes and is known to have limited exposure on the mature virion. Similarly, the AG strand epitope is mostly conserved across DENV serotypes and can be partially occluded by other E protein domains on the mature virion.^19^ In contrast, the lateral-ridge epitope, which spans over the EDI-EDIII linker region, and FG and BC loops are more exposed on the virion surface.

Although mouse immunization with EDIII antigens has been shown to provide immunity against DENV infection, it has been demonstrated that immunization with recombinant EDIII elicited primarily antibodies with moderate to weak neutralization, largely targeting the AB loop epitope.^20^ Examples of mouse anti-EDIII that target the AB-loop are 2H12 and 3E31.^15,21^ On the contrary, most of the potently neutralizing anti-EDIII characterized thus far predominantly target either the AG-strand epitope or the lateral-ridge epitope. Antibodies targeted at the AG-strand epitope, such as 1A1D-2, 4E11, 4E5A, and 513, are cross-reactive and exhibit lower neutralization potency compare to antibodies that target the lateral ridge.^22−26^ Conversely, highly neutralizing antibodies binding to the lateral ridge, such as 2C8 and 3H5, are often type-specific due to sequence variability among the four DENV serotypes. Particularly, 3H5 is a well-characterized murine antibody that binds to and neutralizes DENV-2 at subnanomolar concentrations, yet exhibits unusually minimal ADE.^13,27^ A passive transfer of 3H5 was shown to protect mice from lethal DENV infection without discernible clinal symptoms.^28^ Additionally, blockade of 3H5 epitope has been correlated with serum antibody neutralization of DENV-2-infected/immunized nonhuman primates.^29^ Considering the unique characteristics and properties of 3H5, the selective elicitation of antibodies akin to 3H5 holds promise in providing effective and safe immunity against DENV.

2C8, like 3H5, is a TS murine antibody that binds to the EDIII lateral ridge epitope and shows potent neutralizing activity against DENV-2. An in-depth comparative study between 2C8 and 3H5 revealed that 2C8 causes ADE typically observed in anti-E antibodies, unlike 3H5. The difference in ADE activity of 3H5 and 2C8 is due to their subtle difference in the binding epitopes alongside their binding affinity and topology.^13^ Thus, the designed antigen that aims to elicit 3H5-like antibodies should, ideally, be able to avoid elicitation of 2C8-like and other ADE-associated or weakly neutralizing epitopes.

A glycan-masking strategy utilizes N-glycans as shields to prevent antibody binding to specific epitopes on a given antigen protein.^30,31^ The strategy has been successfully utilized to control antibody recognition across multiple antigens. For example, a glycan-masked version of an HIV vaccine candidate eOD-GT8 was able to focus the antibody response to the targeted CD4-binding site.^32^ In the case of influenza virus, a hyperglycosylated hemagglutinin could shift the antibody response away from a variable head region and toward a more conserved stalk region of the protein.^33^ Similarly, a glycan-masked Zika virus EDIII was able to shield an artificially exposed non-neutralizing epitope, diverting an antibody response toward more strongly neutralizing epitopes.^34^ These examples illustrate the effectiveness of the glycan shield in steering antibodies away from undesired epitopes or focusing them on a desired epitope.

Herein, we employed a glycan-masking strategy to target the epitope of 3H5 on the EDIII of DENV-2. Utilizing a structural-guided approach, we design glycosylated EDIII antigens with shielded nontargeting cross-reactive epitopes (AB loop and AG strand) and the ADE-associated epitope of 2C8. Each N-glycan position was evaluated for its shielding capability against a panel of anti-EDIII antibodies with known binding epitopes. Subsequently, the glycan positions demonstrating selective and effective shielding were combined to afford an antigen that preferentially displayed the 3H5 epitope. This glycosylated antigen served as a bait antigen in an epitope-directed scFv-antibody selection campaign to select antibodies that bind to the targeting epitope of 3H5. The selected antibodies were shown to exhibit potent neutralization with a low ADE similar to the template antibody 3H5. These results suggest the potential of using the glycosylated EDIII antigen as an epitope-focused immunogen to selectively induce 3H5-like antibodies.

## Results and Discussion

### Design and Selection of Glycosylated DENV-2 EDIII

**Figure 2:**
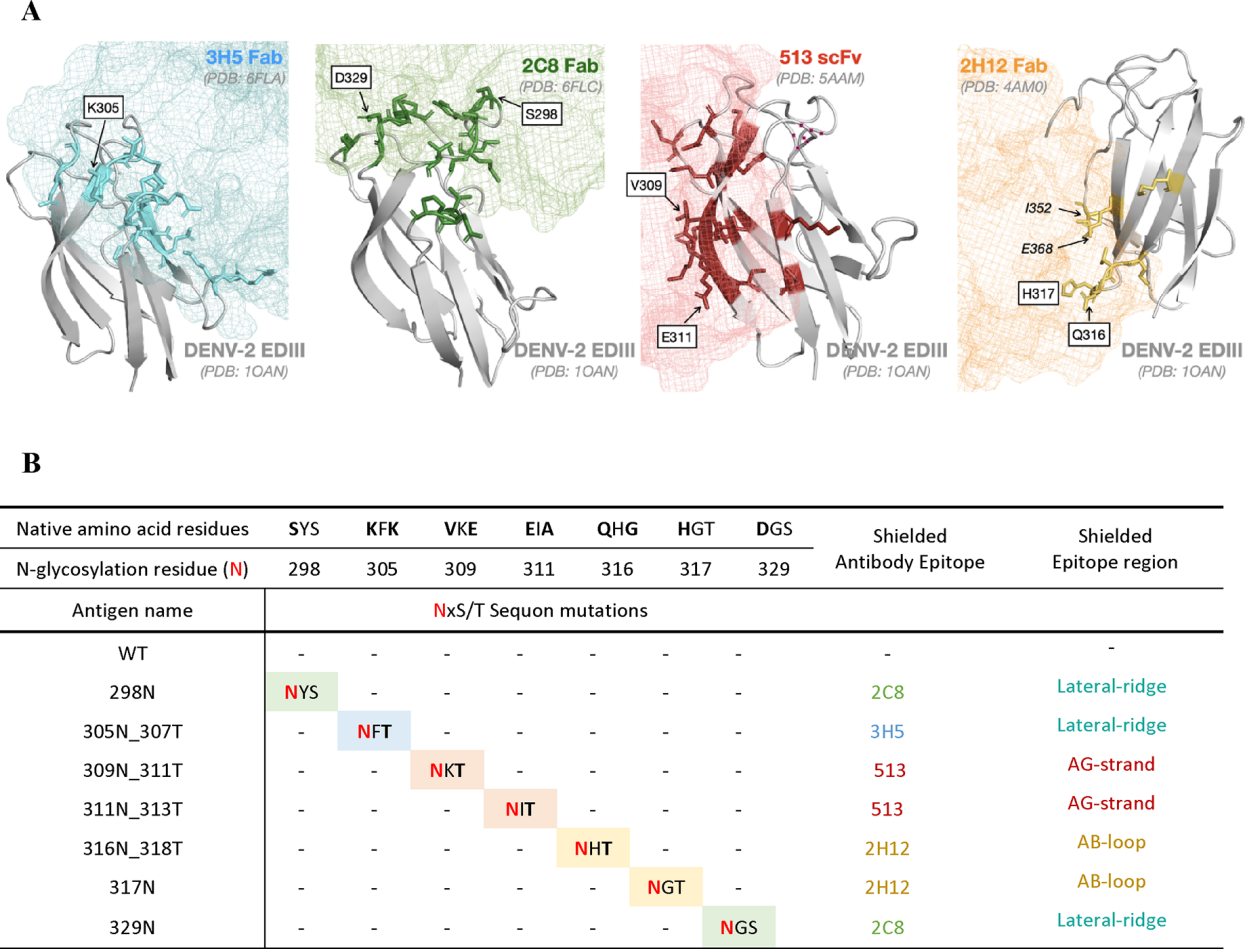
Structure-guided selection of the N-glycosylation sites for selective epitope shielding. (A) Overlayed DENV-2 EDIII structures with an antigen–antibody complex of each template antibody. The selected residues are shown as sticks with labels. (B) Table summary of the selected N-glycosylation sites and shielded epitopes. The native residues of DENV-2 EDIII (amino acids 298–394) subjected to an NxS/T sequon mutation are represented as bold letters. The potential N-glycosylation residues are shown in red.

We initially identified N-glycosylation sites that could selectively shield the nontargeting epitopes—specifically the AB loop, AG strand, and an ADE-associated epitope of 2C8. We assessed the effectiveness and selectivity of each glycan shield with a panel of four template anti-EDIII antibodies. This antibody panel consists of 3H5 and three other antibodies that bind to the three nontargeting epitopes: 2H12, 513, and 2C8 (Figure 1B). The mouse antibody 2H12 binds to a cross-reactive AB loop epitope, while the engineered and humanized antibody 513 binds to a cross-reactive AG strand epitope. 2C8, a mouse antibody, binds to the lateral-ridge epitope with a binding footprint distinct from that of 3H5. A sequence alignment of each antibody’s epitope and crystal structures of the EDIII-antibody complex guided the selection of N-glycosylation sites. Briefly, we identified epitope residues of each antibody allowing the introduction of the NxS/T sequon without mutating other antibody epitopes or potentially causing disruption the secondary structure of EDIII (Figure S1). The residues were mapped onto an unbound DENV-2 EDIII domain to trace their relative positions on the epitope (Figure 2A). For each nontargeting epitope, we selected two mutation sites for experimental determination of N-glycosylation. Additionally, we chose a glycosylation site at residue 305 to shield the targeting 3H5 epitope. This residue was chosen based on a previous report showing that residue K305 serves as a key binding residue of multiple EDIII lateral-ridge targeting antibodies.^35^ We reasoned that an antigen with a glycan shield at residue 305 could be used to quickly identify antibodies binding to the lateral-ridge region of EDIII, potentially including those targeting the 3H5 epitope.

**Figure 3:**
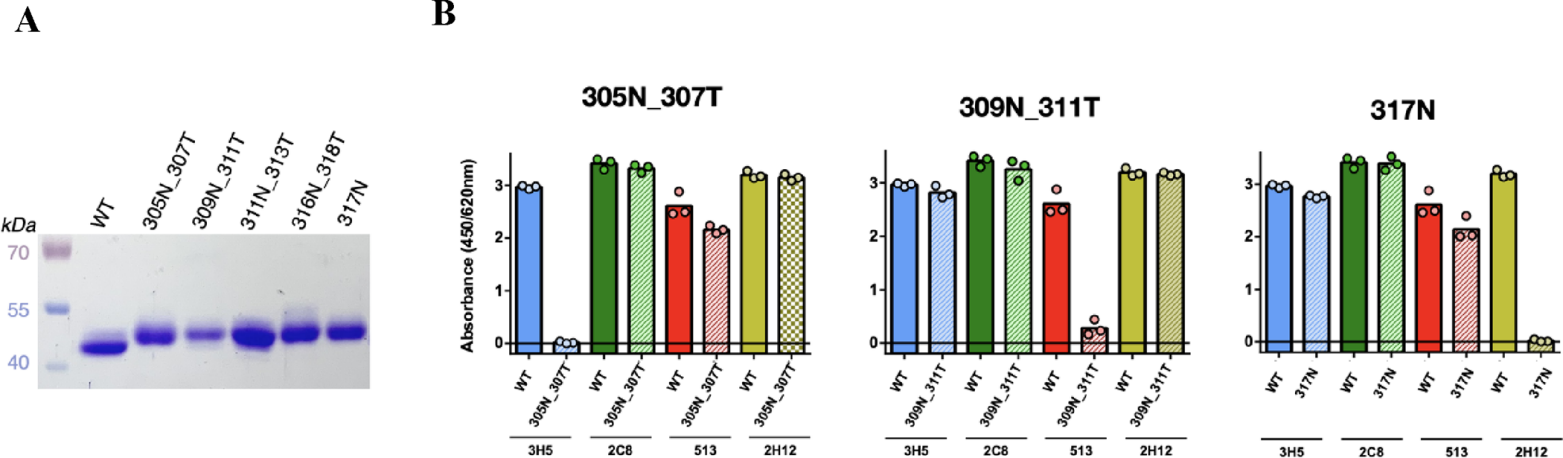
Analysis of purified monoglycosylated EDIII antigens. (A) Apparent size of EDIII antigens on SDS-PAGE under denaturing conditions (reduced and heat) stained with Coomassie blue. (B) Binding of the antibody panel of 3H5, 2C8, 513, and 2H12 to EDIII WT compared with monoglycosylated EDIII antigens (three technical replicates).

Seven N-glycosylation sites (Figure 2B) were selected and individually introduced to a DENV-2 EDIII expression vector using site-directed mutagenesis. The EDIII antigens were expressed as Fc-fusion proteins (EDIII-Fc) to facilitate the expression in 293T cells. Successful antigen expression was confirmed through Western blot analysis of transfected 293T cell lysates. The results showed that most of the mutated antigens appeared larger in size compared to the EDIII wild-type (WT) antigen, except the 298N and 329N mutants designed to shield the 2C8 epitope (Figure S2A). The increased apparent size of EDIII antigens suggested successful N-glycosylation at the mutation site. Subsequently, the five monoglycosylated antigens (305N_307T, 309N_311T, 311N_313T, 316N_318T, and 317N) were purified and evaluated for their binding against the template antibody panel (Figure 3A and Figure S2B).

Out of the five monoglycosylated EDIII, only mutants 305N_307T, 309N_311T, and 317N appear to effectively and specifically shield binding of the antibody panel (Figure 3B). Mutant 305N_307T specifically abrogates the binding of 3H5 while retaining binding to 2C8, 513, and 2H12. Mutant 309N_311T specifically shields 513 binding while maintaining binding to 3H5, 2C8, and 2H12. Lastly, mutant 317N specifically shields 2H12 while maintaining binding to 3H5, 2C8, and 513. Therefore, these three glycosylation mutations were used in subsequent experiments. EDIII mutant 311N_313T and 316N_318T were eliminated from the study during initial testing. Mutant 311N_313T failed to shield 513 binding, while mutant 316N_318T showed reduced binding to 3H5 and 513, potentially suggesting that the N-glycan at residue 316 disrupted the EDIII conformation (Figure S2B).

**Figure 4:**
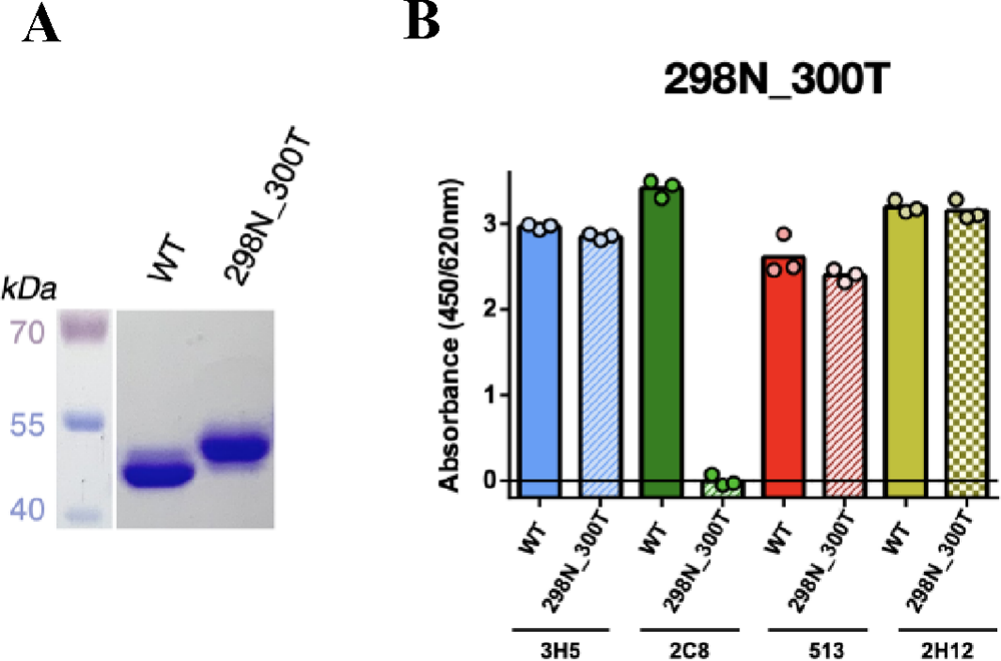
Analysis of purified monoglycosylated EDIII antigen mutant 298N_300T. (A) Apparent size of EDIII antigens on SDS-PAGE under denaturing conditions (reduced and heat) stained with Coomassie blue. (B) Binding of the antibody panel of 3H5, 2C8, 513, and 2H12 to EDIII WT compared with EDIII antigen mutant 298N_300T (three technical replicates).

We then addressed the issue concerning the nonglycosylated antigen 298N and 329N, aimed to shield the 2C8 epitope. Both EDIII mutants relied on native serine residues (S300 and S331) as part of the NxS sequon, which is less efficiently glycosylated compare to the NxT sequon.^32,36,37^ To address this issue, residues S300 and S331 were mutated to threonine, affording EDIII mutants 298N_300T and 329N_331T. In addition, we speculated that the N-terminal position of the residue 298N might lack an appropriate length to be recognized by glycosyl-transferring enzymes and contribute to the lack of glycosylation. As a potential solution, four additional amino acid residues of the EDI-EDIII linker 294–297 (^294^LKGM^297^) were added to the EDIII antigens (Figure S3A). Cell lysates of transfected 293T cells containing these new constructs were compared to those transfected with the original designs. The western blot analysis showed that only the 298N_300T mutation yielded a complete shift in protein size, while the 298N mutant with a longer sequence (298N long) yielded partially glycosylated antigen as reflected by two different sizes of the EDIII antigen. Unfortunately, no shift in protein size was observed for mutant 329N_331T, indicating no glycosylation (Figure S3B). Consequently, mutant 298N_300T was purified and evaluated for its binding with the antibody panel (Figure 4). The ELISA result in Figure 4B indicates that N-glycosylation at position 298 can specifically shield 2C8 binding while maintaining binding to 3H5, 513, and 2H12 antibodies.

Collectively, we have identified that EDIII antigen with mutations 298N_300T, 305N_307T, 309N_311T, and 317N are glycosylated, evident from their apparent size shift compared to the EDIII WT antigen in an SDS-PAGE analysis. Treatment of these EDIII antigens with PNGase F enzyme resulted in proteins of similar apparent sizes consistent with the removal of N-glycans by the enzyme (Figure S5). These monoglycosylated EDIII antigens can selectively abrogate the binding of anti-EDIII antibodies 2C8, 3H5, 513, and 2H12.

**Figure 5:**
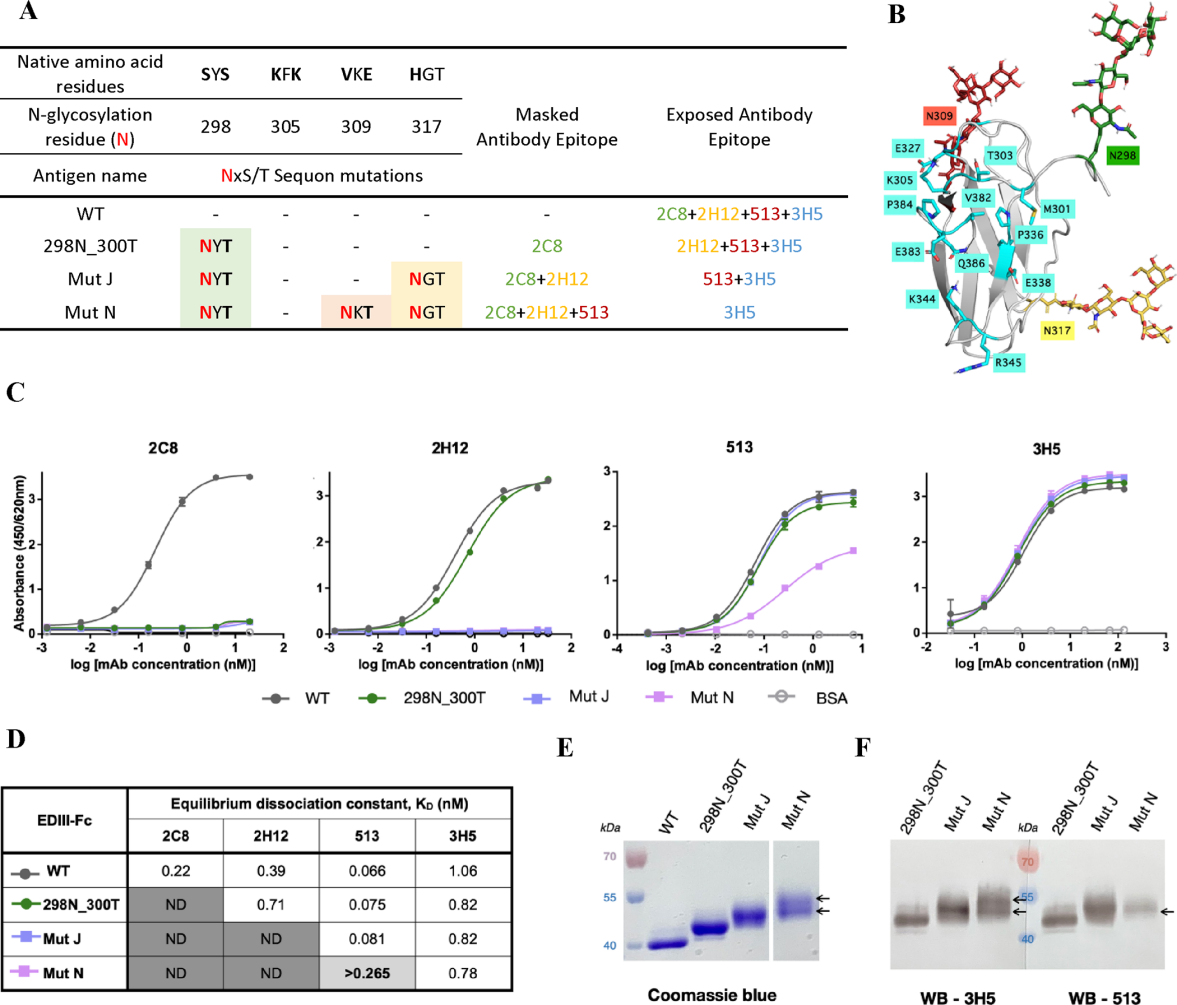
Multiple glycosylation sites on EDIII antigens. (A) Summary table of sequential incorporation of N-glycans onto the EDIII antigen. (B) Model of EDIII Mut N illustrating glycosylation at engineered residues 298N, 309N, and 317N. Epitope residues of 3H5 are displayed in cyan. The model was created from Glycoprotein Builder.^38^ (C, D) Binding of the template antibodies 2C8, 2H12, 513, and 3H5 to EDIII WT compared with EDIII antigens with one, two, and three glycosylation sites (two technical replicates, error bars representing SD of replicates in the same plate. ND = not detected). The *K*_D_ values of each antibody are displayed in panel (D). (E) Apparent size of monoglycosylated 298N_300T, diglycosylated Mut J, and triglycosylated Mut N EDIII antigens on SDS-PAGE under denaturing conditions (reduced and heat) stained with Coomassie blue. The arrows indicate the two overlapping bands of the EDIII Mut N. (F) Binding of EDIII 298N_300T, Mut N, and Mut J to 3H5 and 513 on a western blot assay. The arrows indicate the EDIII N mutant antigen detected by each antibody.

We subsequently introduced multiple glycosylation sites onto an EDIII antigen (Figure 5A,B). The antigens were expressed, purified, and characterized with SDS-PAGE and size-exclusion chromatography in comparison to the WT antigen and monoglycosylated mutant 298N_300T (Figure 5E and Figure S6). A diglycosylated EDIII mutant J (Mut J) combines glycosylation at residue 298N and 317N shows selective shielding of 2C8 and 2H12 while maintaining binding to 513 and 3H5 with similar *K*_D_ values to EDIII WT (Figure 5C,D). A triglycosylated EDIII mutant N (Mut N) designed to selectively present the epitope of 3H5 by employing three N-glycosylation at residues 298N, 309N, and 317N to shield epitopes of 2C8, 2H12, and 513. The EDIII Mut N can effectively maintain binding to 3H5 while selectively shielding binding of 2C8 and 2H12 as demonstrated by ELISA. However, Mut N shows only reduction in binding to 513 as reflected by increased equilibrium dissociation constant (*K*_D_) value, rather than complete shielding. We hypothesized that this binding reduction is caused by an incomplete glycosylation at residue 309N that shields the 513 epitope. This hypothesis stemmed from observing the apparent size of EDIII Mut N on an SDS-PAGE analysis. The antigen appears as two overlapping bands spanning over the expected size of the diglycosylated EDIII antigen Mut J (Figure 5E). The hypothesis was validated by a western blot analysis, which showed that 3H5 antibody could bind to both of the overlapping bands, while the 513 antibody only binds to the lower band of Mut N, which appears to be in a comparable size to a diglycosylated EDIII Mut J (Figure 5F), suggesting that a fraction of EDIII Mut N lacks N-glycan at a mutated residue 309N. Nevertheless, the comparable *K*_D_ values of EDIII Mut N and WT with 3H5 antibody indicate that the targeting epitope of 3H5 is well preserved on EDIII Mut N, while other nontargeting epitopes are shielded, albeit incompletely at residue 309N on the A-strand.

We additionally deglycosylated EDIII Mut N under nondenaturing conditions to examine whether the removal of glycans would restore the binding of template antibodies. Despite the antigen being completely deglycosylated, the binding activities of 2C8, 2H12, and 513 were not restored (Supplementary Figure S7). We reason that this is partly because the mutated amino acids are key binding residues of the template antibodies. In addition, the binding restoration assessed by ELISA might have been confounded by the enzymatic deglycosylation reaction itself, as control experiments with the deglycosylated EDIII WT and Mut N showed reduction in the binding of 3H5 compared to their nondeglycosylated counterparts (Figure S7C).

### Epitope-Directed Selection of Anti-EDIII Targeting the 3H5 Epitope

EDIII Mut N was used as a bait antigen to select antibodies that bind to the targeting 3H5 epitope. This selection was performed with a dengue immune scFv-phage library analogous to the antibody repertoire of dengue patients. The scFv-phage library was constructed from peripheral blood mononuclear cells (PBMCs) samples of 12 dengue patients with secondary infection (three patients for each serotype) collected during acute and convalescence phases. All patients manifested severe dengue hemorrhagic fever (DHF). The scFv-phage selection aimed to assess whether (i) the three glycosylation sites on the EDIII Mut N are sufficient to selectively engage antibodies binding to the targeting epitope of 3H5 and (ii) antibodies that bind to the targeting epitope exhibit potent neutralization activity with unusually low ADE similar to 3H5. These experiments were conducted to evaluate the potential of EDIII Mut N to serve as an epitope-focused immunogen to selectively elicit a response that yields 3H5-like antibodies, i.e., minimal ADE and potent neutralizing activity, as a selective elicitation of such antibodies would need a selective representation of the 3H5 epitope. In addition, the minimal ADE with potent neutralization character of these 3H5-like antibodies needs to be confirmed.

**Figure 6:**
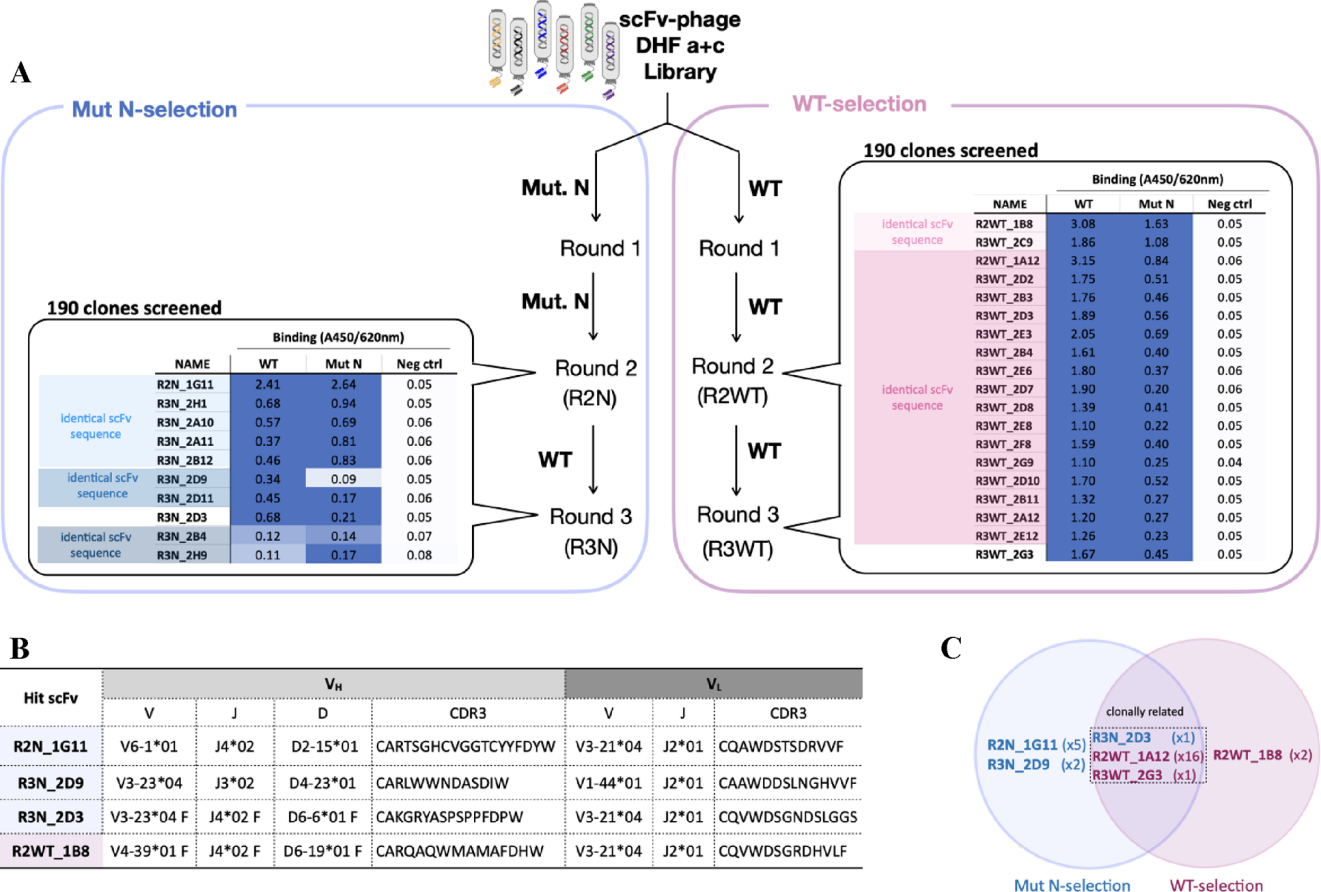
scFv-phage selection campaigns. (A) Selection scheme of the Mut N-selection and WT-selection. Hit clones from each selection and their binding to EDIII antigens (WT or Mut N) and a negative control (Neg ctrl is an unrelated Fc-fusion protein). Hit clones with identical scFv sequence from each selection are highlighted in the same color. (B) Immunoglobulin germline gene analysis of each distinct hit scFv. (C) Venn diagram summarizing distinct scFv hits (reactive to both EDIII WT and Mut N) from the two selection campaigns. The number of times the clone was identified as a hit are indicated in the parentheses following the clone names.

Three selection rounds were performed with EDIII Mut N (Mut N-selection, Figure 6A). A total of 190 monoclonal phages were randomly picked after the second and third selection rounds (95 clones for each round) for phage ELISA screening. From these, 12 hit clones showing binding to both EDIII Mut N and WT antigens were identified. The scFv sequences of these hits revealed four distinct scFvs, namely, R2N_1G11, R3N_2B4, R3N_2D3, and R3N_2D9 (referred to as 1G11, 2B4, 2D3, and 2D9, respectively) (Figure 6B). The frequency of each distinct clone identified in Figure 6C indicated a higher enrichment of clone 1G11 (identified 5 times) compared to clone 2D9, 2B4, and 2D3 (identified 2, 2, and 1 time, respectively). We noted that the first two rounds of the selection used EDIII Mut N as a bait antigen, while the third selection round used EDIII WT as a bait antigen to mitigate the possibility that the EDIII Mut N may carry any unintentionally created/artificial epitopes that were not presented in the WT antigen. This was speculated due to most output phage clones from the second selection round bind to EDIII Mut N do not bind to EDIII WT (Figure S8). However, most of these clones were later validated to be true false positive, i.e., showing negative binding result upon re-examination, containing incomplete scFv sequence or cannot read the phagemid sequence. We reasoned that this outcome was due to the specificity of the EDIII Mut N that selectively enriched mostly 3H5-epitope binders, which are expected to exist in the library in a very small fraction. After only two selection rounds, a number of false positive clones were picked up during screening since the true hits have not been sufficiently enriched. Additionally, we have set a low the cutoff (absorbance > 0.14), which could contribute to the high false positive rate observed.

To directly compare the selectivity of EDIII Mut N as the bait antigen, another selection campaign was performed in parallel using only EDIII WT as the bait antigen (WT-selection; Figure 6A). This selection yielded a total of 19 hit clones from 190 clones randomly picked from the second and third rounds of the selection (95 clones for each round). The scFv sequence of hit clones from the WT-selection revealed three different scFv sequences, namely, R2WT_1A12, R2WT_1B8, and R3WT_2G3 (referred to as 1A12, 1B8, and 2G3, respectively). The frequency of each distinct clone showed a higher enrichment of clone 1A12 (identified 16 times) compared to clones 1B8 and 2G3 (identified 2 and 1 time, respectively, Figure 6C).

An IMGT-V/QUEST analysis of the distinct scFvs from both Mut N-selection and WT-selection is shown in Figure 6B. The analysis showed that scFv 1G11, 2D9, 2B4, and 1B8 utilize different combinations of V, D, and J genes in their heavy chain variable regions and different combinations of V and D genes in their light chain variable regions. However, 2D3 (from Mut N selection), 1A12, and 2G3 (both from WT-selection) are clonally related with identical variable regions of the heavy chain (V_H_) sequence and 94–95% similarity in variable regions of the light chain (V_L_) sequence (Figure S9). Therefore, only R3N_2D3 was further used in this study. All distinct hit clones from the two selection campaigns were subsequently verified for their reactivity toward a soluble DENV-2 envelope protein (D2E80) and DENV-2 virion. All clones show reactivity to the D2E80 and DENV-2 virion except clone 2B4. The clone was shown to be a nonspecific binder, and thus 2B4 was dropped from the study (Figure S10). Additionally, further attempts to screen more hits from Mut N-selection resulted in an identification of duplicates of 1G11, highlighting the preference of the Mut N-selection toward this particular clone (Figure S11).

**Figure 7:**
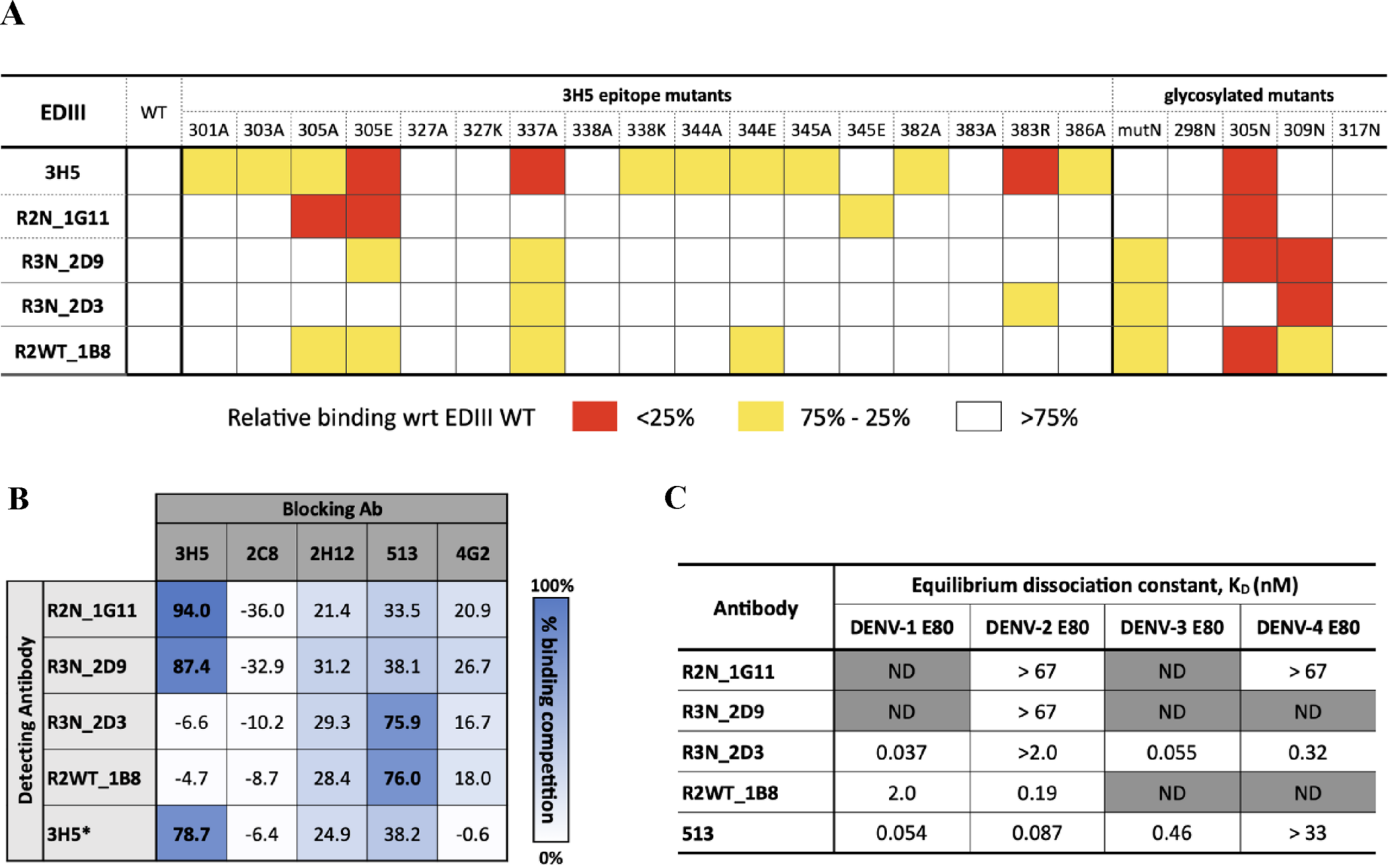
Epitope mapping by mutagenesis on 3H5 epitope. (A) Epitope mapping by point mutagenesis on 3H5 epitope residues. Binding residues of each antibody are defined point mutation that leads to either severe or moderate binding reduction. Mutations with % relative binding less than 25% are defined as severe binding reduction while mutations with the % relative binding from 75 to 25% are defined as moderate binding reduction. (B) Binding of scFv-derived antibodies to D2E80 in the presence of template antibodies (Competitive ELISA). An anti-E antibody 4G2 that binds to domain II of the DENV envelope protein was used as a negative control. 3H5* detecting antibody was a chimeric 3H5 for all competition assays except for an experiment with 513 where a mouse 3H5 antibody was used. (C) Equilibrium dissociation constant (*K*_D_) of scFv-derived antibodies measured with indirect ELISA using soluble E80 protein of four DENV serotypes (the representing values are the average of two technical replicates). A control anti-EDIII antibody 513, which is cross-reactive, showed *K*_D_ values in line with literature reported values.^39^

The four distinct phage clones, 1G11, 2D9, 2D3, and 1B8, underwent testing to determine if they utilized binding residues within the targeting 3H5 epitope. A panel of individually mutated EDIII antigens at the 3H5 epitope residue (3H5 epitope mutants) was used in combination with a panel of monoglycosylated EDIII antigens (glycosylated mutants). The binding residue of each scFv-phage was defined as a point mutation resulting in a binding decrease lower than 75% relative to the EDIII WT. The result revealed that all four scFv-phage share binding residues with 3H5, albeit at different residues, and the mutations resulted in decrease in binding to various extents (Figure 7A and Figure S12). 1G11 showed severe binding reduction (<25% binding compared to EDIII WT) to all mutations at position 305, while the 345E mutation only moderately affected its binding (75–25% binding compared to EDIII WT). Glycosylation at 305N severely reduced 2D9 binding, while mutation 305E and 337A moderately reduced its binding. In addition, a binding reduction was also observed with EDIII Mut N and 309N_311T, both carrying N-glycosylation at residue 309N. Similarly, 2D3 showed binding reduction to antigen Mut N and 309N_311T, akin to 2D9, along with moderate binding reduction with mutations 337A and 383R. Lastly, 1B8 showed binding reduction to all mutations at position 305, while mutation 337A, 344E, Mut N, and 309N_311T caused moderate binding reduction. Notably, residue 305 appeared to be a critical binding residue for three out of four hit scFv-phage clones; 1G11, 2D9, and 1B8. Residue K305 is a key epitope residue of 3H5 and several other lateral-ridge targeting antibodies; therefore, we reasoned that these scFv-phage clones bind to the lateral-ridge region of EDIII, potentially binding at or near the targeting epitope of 3H5.

To further distinguish the binding epitopes of the scFv-phage, the V_H_ and V_L_ sequences of each scFv were cloned into mammalian expression vectors of human IgG1 antibody and expressed as a full-length human antibody. The reactivities of the purified scFv-derived IgG1 antibodies to virion of all four DENV serotypes and Japanese encephalitis virus (JEV) were confirmed with a virion capture ELISA (Figure S13A,B). The antibodies were then assessed for their binding to soluble DENV-2 E protein (D2E80) in the presence of template antibodies using a competitive ELISA. The results in Figure 7B revealed that 3H5 competes with 1G11 and 2D9, suggesting that these antibodies bind to an epitope similar epitope as 3H5. On the contrary, 3H5 does not compete with 2D3 and 1B8, suggesting that these antibodies bind to distinct epitopes from 3H5. Instead, both 2D3 and 1B8 compete for binding with 513, which targets the AG strand epitope, known to be highly overlapping with the lateral-ridge epitope and its tendency for cross-reactivity.

The scFv-derived antibodies were additionally assessed for their binding affinity using soluble E80 of all four DENV serotypes with an indirect ELISA (Figure 7C and Figure S13C). Consistent with the virion capture ELISA, 1G11 binds to E80 of DENV-2 and DENV-4, while 2D9 shows type-specific reactivity toward DENV-2 E80. We noted that the highest concentration of 1G11 and 2D9 used in the assay was ∼67 nM (10 μg/mL), which could not reach binding saturation under the assay conditions. Antibody 2D3 shows subnanomolar *K*_D_s across the four serotypes, in agreement with its reactivity in a virion capture ELISA. However, 1B8 exhibits subnanomolar *K*_D_s with only DENV-1 and DENV-2 E80 (Figure 5C), while the virion capture ELISA showed that 1B8 also binds to DENV-3 and weakly binds to DENV-4 (Figure S13B). This indicates that 1B8 is a cross-reactive antibody with a stronger binding affinity toward DENV-1 and DENV-2 and a weaker binding affinity toward DENV-3 and DENV-4. These findings further support that 1G11 and 2D9 bind to a more type-specific lateral-ridge epitope, highly similar to 3H5, while 2D3 and 1B8 bind to a more cross-reactive AG strand epitope. We speculate that the cross reactivity of 1G11 (DENV-2 and DENV-4) might stem from K305 being a critical binding residue present in both DENV-2 and DENV4 EDIII.

Additionally, we further demonstrated the specificity of the Mut N-selection by characterizing binding of all distinct phage output from the WT-selection that showed specific binding to EDIII WT but not to Mut N (EDIII WT-specific phage). A total of 11 distinct scFv-phages was identified and subsequently assessed for their binding with a panel of EDIII antigens. Each monoglycosylated EDIII (298N_300T, 305N_307T, 309N_311T, and 317N) serves as a probe to roughly map the binding region of these scFv-phage (Figure 2B). Interestingly, all 11 phage clones show very little to no binding to EDIII mutant 309N_311T, which represents the AG-strand epitope (Figure S14). This suggests the AG-strand as their putative targeting epitope. We noted that two clones, namely, R2WT_1E3 and R3WT_2A1, were mischaracterized by the initial phage screening and they actually bind to both EDIII WT and Mut N upon reassessing for binding. Nevertheless, this result directly demonstrates that WT-selection only enriched nonlateral ridge targeting scFv-phage, specifically the AG strand epitope.

Collectively, the results demonstrated the ability of EDIII Mut N to selectively engage with scFv-phage binding to the lateral-ridge region of DENV-2 EDIII, including the 3H5 epitope. Hit clones from Mut N-selection, 1G11, 2D3, and 2D9 all share binding residues with 3H5. A strong preference toward clone 1G11 is evident by the higher frequency of identification compared to 2D3 and 2D9. The lower frequency of finding 2D3 and 2D9 might be due to their reliance on the amino acid 309 as a binding residue, which was less enriched by the selection with an incompletely glycosylated residue at 309N of EDIII Mut N. A competitive ELISA further differentiated the binding epitopes of 1G11 and 2D9 from 2D3. While 1G11 and 2D9 compete with 3H5, suggesting their similar binding epitopes, 2D3 does not compete with 3H5, suggesting their different epitopes from 3H5. Instead, 2D3 competes with 513 for an adjacent AG strand epitope. Importantly, none of the hit clones from Mut-N selection were found to bind outside the epitopes defined by the 4 template antibodies, suggesting that the three glycosylation sites of EDIII Mut N are sufficiently covering all epitopes within the dengue immune scFv libraries. A direct comparison of hit clones from the Mut N-selection and WT-selection further supports the ability of Mut N to selectively present the 3H5 epitope. The hit clones from the WT-selection, namely, 1B8, 1A12, and 2G3, bind to a cross-reactive AG epitope. Additionally, all EDIII WT-specific phages from WT-selection do not bind to EDIII 309N_311T, suggesting the AG strand as their probable epitope. We hypothesize that the enrichment of cross-reactive AG-strand binders in WT selection could be due to the abundance of these phages in the library originating from secondary DHF patients. Chaudhury et al. (2017) showed that antibodies from secondary DENV patients exhibit a higher propensity of being cross-reactive anti-EDIII antibodies than those from patients with primary infection.^40^ Furthermore, the high washing stringency employed during phage selection (0.1% Tween-20 in PBS, 20 washes) might influence the enrichment of high affinity AG-strand binders, as inferred from the two characterized AG-strand binders 2D3 and 1B8. Only Mut N-selection could select for scFvs binding to the 3H5 epitope such as 1G11 and 2D9, which also exhibit apparently lower affinities as reflected by their higher *K*_D_ values compared to the AG binders, 1B8 and 2D3. This highlights the advantage of using an epitope-focused bait antigen to select for desired antibodies, which would have otherwise been over-dominated by more prominent or higher affinity undesired antibodies present in the library pool. Despite the selective enrichment by EDIII Mut N, we observed a high false positive rate in the screening of the second-round Mut N-selection. This might be due to a combination of the EDIII Mut N’s selectivity toward the 3H5 epitope and a potentially low abundance of scFv-phage that binds to the 3H5 epitope initially present in the scFv-phage library. EDIII is a subdominant domain of the E protein; therefore, a smaller population size of antibodies that target only a certain region of a EDIII is expected.

### scFv-derived Antibodies Are Potently Neutralizing and Minimally Enhancing Infection in DENV-2

**Figure 8:**
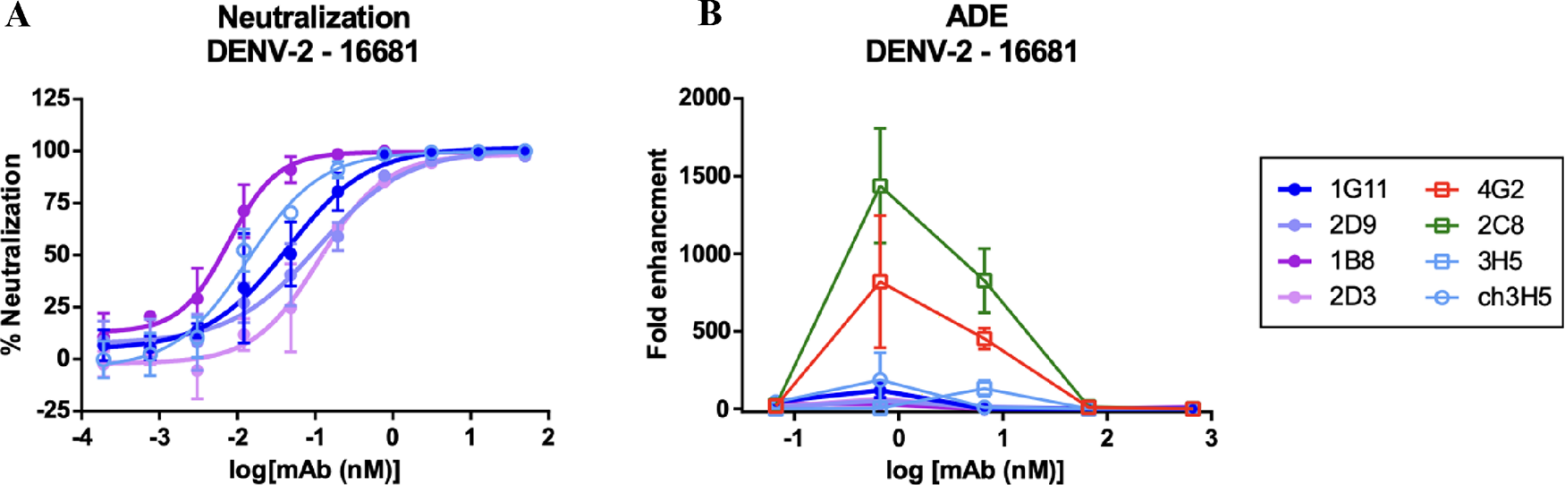
Neutralization and antibody-dependent enhancement (ADE) of the scFv-derived anti-EDIII mAbs. (A) Neutralization of scFv-derived IgG1 in comparison with chimeric 3H5(ch3H5) antibody. (B) ADE of the scFv-derived antibodies in comparison with 4G2, 2C8, 3H5, and ch3H5 antibodies.

A focus reduction neutralization test (FRNT) showed potent neutralization of DENV-2 strain 16681 by all four scFv-derived antibodies with 50% neutralization titer (FRNT-50) in a subnanometer range (1G11:0.044 nM, 2D9:0.110 nM, 2D3:0.115 nM, 1B8:0.008 nM, Figure 8A). These titers are similar to a chimeric 3H5 with human IgG1 constant regions (0.014 nM). Additionally, the ADE assay of these scFv-derived antibodies in a monocytic cell line U937 demonstrated minimal to no enhancement, similar to 3H5 antibodies. Conversely, the 2C8 and 4G2 antibodies showed prominent ADE activity typically observed in anti-E antibodies in the same assay (Figure 8B).

These results suggest that 1G11 and 2D9, which bind to a similar epitope to 3H5, are indeed highly potent neutralizing antibodies with minimal ADE. Meanwhile 2D3 and 1B8, which bind to the adjacent AG strand epitope, are also potent neutralizing antibodies with minimal ADE. One of the potential factors that contribute to low ADE activity of 3H5 is its engagement to the CD loop residues, specifically K344 and R345, which located near the viral membrane and could result in a “flat” laying binding.^13^ Residue K344 was shown to mediate binding of 1B8, and it might help explain its low ADE activities despite its distinct binding epitope. However, the observed minimal to no ADE activity of these antibodies likely involves multiple factors. Therefore, further investigation into the contributing factors to the observed low ADE activity is warranted.

In summary, we have detailed the utilization of a glycosylation shield on DENV-2 EDIII, enabling the discovery of new highly potent neutralizing antibodies with minimal ADE properties through an epitope-directed scFv-phage selection campaign. Glycan positions on the EDIII antigen bait could be chosen to achieve high specificity to a particular epitope while leaving the other epitopes undisturbed. We combined three glycan shields to create an EDIII Mut N antigen that preferentially displays the targeting epitope of 3H5, an unusually highly potent neutralizing antibody with minimal ADE. The Mut N-selection, using EDIII Mut N as a bait antigen, proved to be selective to the 1G11 antibody that binds to the targeting epitope. However, some antibodies were identified at a lower frequency. These less frequently identified antibodies appear to bind to the adjacent AG strand epitope and are likely enriched during phage selection due to incomplete glycosylation at residue 309N of EDIII Mut N antigen. We speculate that the lower N-glycan occupancy at residue 309N might be due to its close proximity to the glycosylation site at either residue 317N or 298N, similar to what has been observed in a study with an HIV envelope SOSIP antigens.^41^ This observation demonstrates a limitation of epitope shielding by engineered N-glycans, where not every amino acid position could be glycosylated to the same level of occupancy. Additionally, certain amino acid mutations are impermissible for N-glycosylation, or the added glycan might disrupt the native conformation of the antigen as observed in EDIII mutation 329N, 329N_331T, and 316N_318T. A complementary technique such as a chemical conjugation of the polyethylene glycol (PEG) moiety to shield epitopes might be needed to achieve a more complete epitope shielding for a given antigen.^42,43^ One caveat on using a PEG chemical conjugation to shield antibody epitopes is that the conjugating moiety would be more uniform and the shield itself (e.g., PEG) could potentially become the target of an antibody (e.g., anti-PEG^44^). In contrast, N-linked glycan composition is nonuniform. The heterogeneity of glycan composition could potentially work in favoring antibody shielding as it would less likely be targeted by an antibody. Furthermore, the shielding effect of glycans has been suggested by a molecular dynamics study to be insensitive to the glycan heterogeneity.^45^ As a bait antigen for antibody discovery, the occupancy of the glycan (how complete the position is glycosylated) might be more important than the composition of the glycan (what is being glycosylated). However, as an immunogen, more studies are needed to elucidate the effects of glycan composition on not only a humoral response, but also a cellular response, as protein glycosylation influences the proteolytic cleavage to generate MHC-associate peptides.^46^

While Mut N selection showed selective enrichment for binding of scFv to the targeting epitope, a parallelly performed WT-selection failed to identify binding of scFv clones to the targeting epitope. This emphasizes the advantage of using a shielded antigen in an epitope-directed selection campaign. Despite the discrepancies and similarities in binding epitopes, all four hit antibodies from both Mut N and WT selections appear to be highly neutral against DENV-2 16681 with a low ADE activity. These results suggest that the unusual properties of 3H5 might exist in the DENV-infected patients antibody repertoire, and a specific elicitation of more 3H5-like antibodies may provide a more efficacious and safer protection. Since Mut N was shown to specifically engage with existing 3H5-like antibodies in the DENV-immune phage library, it would be interesting to examine whether an animal model immunization with Mut N could induce 3H5-like antibodies. As an alternative, a selection campaign with a DENV-naïve antibody library may serve as a proxy to suggest the propensity of the inducing anti-EDIII antibodies if immunization were to happen in DENV-naïve individuals/animals. Additionally, the characterization of two AG-strand binders with low ADE, 1B8 and 2D3, suggests that the low ADE characteristic of 3H5 is not strictly confined to its exact epitope but more likely to the antigenic regions of lateral-ridge and AG strands. Further comparative studies on more lateral-ridge and AG-strand targeting antibodies may shed light on this observation. Lastly, since some of the selected antibodies are cross-reactive, the neutralization and ADE activities with the other three serotypes will be investigated and reported in due course. The factors underlying the observed low ADE of these antibodies warrant further investigation to help understand the molecular basis of these minimal to no ADE anti-EDIII that has been described in only a few literature studies to date.^13,15,21,47^

## Methods

### Cell Lines and Virus

Human embryonic kidney cells 293T were cultured in a DMEM medium. Vero cells were cultured in MEM medium. Human monocytes U937 were cultured in an RPMI 1640 medium. All culture media were purchased from Gibco and supplemented with 10% (v/v) heat-inactivated FBS (HyClone or Gibco), 1× Penicillin-G-Streptomycin (Gibco), and 1× GlutaMAX (Gibco). Expi293F cells were cultured in Expi293 Expression Medium (ThermoFisher Scientific) and maintained according to the manufacturer’s instructions. Dengue virus serotype-1 (DENV-1, Hawaii), DENV-2 (16681), DENV-3 (H87), DENV-4 (H241), and JEV (Nakayama) were propagated in C6/36 cells cultured in Leibovitz-15 medium (Gibco), supplemented with 3% heat-inactivated FBS (Gibco) and 10% tryptose phosphate broth (sigma-Aldrich)

### Cloning, Expression, and Protein Purification

#### Antibodies

3H5 (IgG1), 2C8 (IgG2a), and 4G2 (IgG2a) antibodies were purified from the hybridoma culture supernatant using HiTrap protein-G HP columns (Cytiva). An anti-E antibody 4G2 was a gift from Armed Forces Research Institute of Medical Sciences (AFRIMS).^48,49^ The 2H12 (IgG2b) antibody was generously provided by Dr. Juthathip Mongkolsapaya (Nuffield Department of Medicine, Oxford University).^21^ The coding sequences for V_H_ and V_L_ genes of antibody 513, 4G2, and h38C2_Arg were synthesized or amplified from a hybridoma cell line and subsequently cloned into pVITRO1 expression vector and were expressed in Expi293F cells.^39,50−52^ The recombinant antibodies were purified with protein A-agarose beads (Sigma-Aldrich).^29^ scFv-derived antibody expression vectors were constructed from the variable regions of the selected phage clones. Briefly, the variable regions, V_H_ or V_L_, were PCR amplified and cloned into an expression vector containing the constant regions of the human IgG1 heavy or light chain. The antibodies were transiently expressed in 293T cells using linear PEI 25K (Polysciences, Taiwan) and purified from cell culture supernatant using protein A-agarose beads (Sigma-Aldrich). All purified antibody concentrations were measured with absorbance at 280 nm using a Nanodrop spectrophotometer. The concentration of 2H12 antibody in a hybridoma culture supernatant was measured using capture ELISA as previously described, using an in-house purified mouse IgG2b as a standard protein.^53^ The concentration of ch4G2 and h38C2_Arg antibodies in transfected 293T culture supernatant was measured using capture ELISA (Capture Ab: polyclonal rabbit antihuman antibody gamma-chain specific (A0423, Dako); secondary Ab: a polyclonal rabbit antihuman antibody gamma-chain specific conjugated with HRP (P0214, Dako)). An in-house purified human IgG1 antibody was used as a standard protein.

#### Antigens

EDIII of DENV-2 16681 was PCR amplified from pMT-D2E80 (unpublished data) and cloned into an expression vector expressing human Fc region with a hexahistidine tag at the C-terminus. Mutations of the antigen were introduced by conventional PCR site-directed mutagenesis. All oligonucleotides were purchased from Macrogen Inc. (South Korea). The EDIII antigens were expressed as Fc-fusion proteins in 293T cells using linear PEI 25K (Polysciences, Taiwan). The antigens were purified from cell culture supernatant of transfected 293T cells using a cobalt-immobilized beads column (TALON) following the instruction manual. The concentrations of the purified antigens were measured by using the Pierce BCA assay (ThermoFisher Scientific). The purity of all purified proteins was assessed by SDS-PAGE followed by Coomassie blue staining.

### Structural Analysis of EDIII and Anti-EDIII Antibodies

The epitope residues of template antibodies, 3H5, 2C8, 513, and 2H12, were mapped onto the amino acid sequence of DENV EDIII to identify potential sites for introducing a sequon (NxS/T) mutation. Specifically, we selected epitope residues unique to each template antibody that would allow mutation without affecting the other antibody epitopes. These residues were mapped onto a DENV-2 EDIII structure extracted from the soluble envelope protein (PDB code: 1OAN) and aligned with an antibody-EDIII complex structure of each antibody (PDB codes: 6FLA, 6FLC, 4AM0, and 5AAM) to yield Figure 2A. For each nontargeting epitope (2C8, 513, and 2H12), we chose two residues located at the epitope center to experimentally determine for N-glycosylation. As for shielding of the 3H5 epitope, residue K305 was chosen as an N-glycosylation site. The decision was based on the residue’s relatively central position and also its critical role as a common residue for multiple lateral-ridge binding antibodies, including 3H5.^35^

### Western Blot

#### Cell Lysate

Cell lysate of transfected 293T cells was prepared using RIPA buffer (ThermoFisher Scientific) according to the manufacturer’s instruction. The total lysate protein was quantified using the Pierce BCA assay (ThermoFisher Scientific). 50 μg of the total lysate was subjected to an SDS-PAGE under denaturing conditions (reduced/heat). The proteins from the SDS-PAGE was transferred onto a nitrocellulose membrane, which was then blocked with 5% skim milk in PBS. The antigen bands were directly probed with goat antihuman IgG (H&L) conjugated with horse radish peroxidase (HRP) (AP112P, Thermo Fisher Scientific) at dilution 1:1000 or probed with the following pairs of primary (*pri*) and secondary (*sec*) antibodies: *pri*-1:500 mouse anti-GAPDH (Santa-Cruz Biotechnology)//*sec-*1:2000 antimouse Ig conjugated with HRP (P0260, Dako) or *pri-*a mixture of anti-EDIII antibodies (in house, hybridoma and transfect 293T supernatant)//*sec*-a mixture of 1:2000 antimouse Ig conjugated with HRP (P0260, Dako) and 1:4000 goat antihuman kappa light chain conjugated with HRP (A18853, Thermo Fisher Scientific). Protein bands were visualized using Clarity Western ECL substrate (Bio-Rad) on an ImageQuant 800 (Cytiva).

#### Purified EDIII Antigens

50 ng of the purified antigens was subjected to an SDS-PAGE under denaturing conditions (reduced/heat) and subsequently transferred onto a nitrocellulose membrane as described above. The antigens were probed with a primary antibody 3H5 (as a hybridoma culture supernatant) or 513 (as a transfected 293T culture supernatant) followed by an appropriate secondary antibody 1:2000 antimouse Ig conjugated with HRP (P0260, Dako) or 1:4000 goat antihuman kappa light chain conjugated with HRP (A18853, Thermo Fisher Scientific). Bands were visualized by a diaminobenzidine (DAB) substrate.

### Size-Exclusion Chromatography (SEC)

Size-exclusion chromatography was performed on AKTA pure (Cytiva) equipped with a Superdex 200 Increase 10/300 GL column (GE Healthcare). The solvent system was 10 mM sodium phosphate buffer with 137 mM sodium chloride and 27 mM potassium chloride (pH 7.4). The chromatography was run at a flow rate 0.75 mL/min over 1.5 column volumes (CV, ∼ 36 mL). The samples were injected into the column at 100uL (diluted to 0.4–0.5 mg/mL). The absorbance at 280 and 210 nm was monitored. Output data were processed in Unicorn Evaluation (version 7.6). The percentage peak integration was calculated from peak absorption at 280 nm.

### Deglycosylation of the EDIII Antigens

1 μg of each EDIII antigen was treated with 1 μL of PNGase F (NEB) under denaturing conditions or 5 μg of EDIII antigen was treated with 2 μL of PNGase F under nondenaturing conditions as per the manufacturer’s instruction. The samples were directly subjected to further analyses (SDS-PAGE, western blot, or antibody ELISA) without purification.

### ELISA

#### General Procedure

Maxisorp ELISA microplates (ThermoFisher Scientific) were coated with an antigen or a capture antibody at 4 °C overnight. Plates were then washed with PBS and blocked with a specified blocking solution at 37 °C for 1 h. For capture ELISA, the antigen was added and incubated at 37 °C for 1 h. The plates were washed and incubated with a primary antibody at 37 °C for 1 h. After washing, the plates were incubated with a secondary antibody at 37 °C for 1 h. Plates were thoroughly washed, and the signal was developed using 50 μL of 1-Step Ultra TMB substrate solution (ThermoFisher Scientific) and stopped with 50 μL of 2 N sulfuric acid. The ELISA signal was immediately measured with an ELISA reader (TECAN Sunrise) at 450/620 nm.

Antibody ELISA: The ELISA plates were coated with 100 ng/100 μL/well of EDIII antigen, except for 2C8, wells were coated with 500 ng/100 μL/well. Plates were subsequently blocked with 1% BSA in PBS and incubated with the anti-EDIII primary antibody. The secondary antibody was either a rabbit antimouse Ig conjugated with HRP (P0260, Dako) at 1:2000 dilution in 1% BSA/PBS or a goat antihuman kappa light chain conjugated with HRP (A18853, Thermo Fisher Scientific) at 1:4000 dilution in 1% BSA/PBS.

#### Phage ELISA Screening

The procedure was modified from monoclonal phage ELISA of the Tomlinson I+J human scFv library. Briefly, the ELISA plates were coated with 50 ng/50 μL/well of EDIII antigen and subsequently blocked with 2% skim milk in PBS. A 40% (v/v) scFv-phage supernatant in 2% skim milk was used as a primary antibody. The secondary antibody was anti-M13 conjugated with HRP (GE Healthcare) at a 1:2500 dilution in 2% skim milk. A “hit clone” was defined as a phage clone that contains a complete scFv sequence and showed reactivity toward EDIII WT and Mut N antigens, but not an unrelated Fc-fusion protein.

#### Phage Capture ELISA for Binding Residue Mapping

The ELISA plates were coated with a polyclonal rabbit antihuman antibody gamma-chain specific (A0423, Dako) and subsequently blocked with 2% skim milk. Each mutant of EDIII antigen at 200 ng/mL (100 μL) was then captured onto plates. A each scFv-phage supernatant at a predetermined dilution in 2% skim milk was used as a primary antibody. The secondary antibody was anti-M13 conjugated with HRP at 1:2500 dilution (GE Healthcare) in 2% skim milk. A control experiment was performed using a polyclonal rabbit antihuman antibody gamma-chain specific conjugated with HRP (P0214, Dako) at 1:6000 dilution in 2% skim milk to ensure a comparable loading of each antigen mutant.

#### Antibody Capture ELISA

The ELISA plates were coated with a mouse anti-E antibody 4G2 at 5 μg/mL (100 μL/well) and subsequently blocked with 1% BSA in PBS. DENV1–4 virus stock (DENV-1 Hawaii, DENV-2 16681, DENV-3 H87, DENV-4 H241, and JEV Nakayama) was diluted to 1:4 dilution in 1% BSA and captured onto plates. After washing, a primary antibody solution of anti-EDIII at 5 μg/mL was added to plates. The secondary antibody was a polyclonal rabbit antihuman antibody gamma-chain specific conjugated with HRP (P0214, Dako) at 1:6000 dilution.

Competitive ELISA: The ELISA plates were coated with 50 ng/50 μL/well of DENV-2 E80 antigen. Plates were subsequently blocked with 1% BSA in PBS and incubated with a blocking Ab (100 μg/mL for 3H5,2C8, and 4G2, 50 μg/mL for 513 and 25 μg/mL for 2H12) or BSA (no competition) for 1 h. The detecting Ab was then added to the well without removing the blocking Ab and incubated for an additional 1 h. The secondary antibody for human or chimeric detecting Ab was a goat antihuman Ig lambda-chain specific conjugated with HRP (A506P, Merck) or a goat antihuman kappa light chain conjugated with HRP (A18853, Thermo Fisher Scientific). The secondary antibody for mouse 3H5 antibody was a rabbit antimouse Ig conjugated with HRP (P0260, Dako). The percentage binding competition is calculated from the following eq 1;
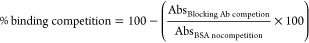
1

### Dengue Immune scFv-Phage Libraries

A dengue immune scFv-phage DHF.c library was constructed from convalescent sera of dengue patients as previously described.^54^ Additionally, a library DHF.a library was constructed from acute peripheral blood mononuclear cells (PBMCs) following the same protocol used for the DHF.c library, using the same set of patients. Twelve PBMCs were collected at the acute phase (day −1) from DENV infected patients in Khon Kaen and Songkhla hospitals during 2004–2009 and kept frozen. All research on humans was approved by the Siriraj Institutional Review Board (protocol number 632/2559), Khon Kaen Hospital Institute Review Board in Human Research (protocol number KE60108), and ethical committee of Songkhla Hospital (protocol number 11/256). Written informed consents were obtained from all subjects.

### scFv-Phage Selection (Biopanning)

scFv-phage selection was conducted following the protocols of Tomlinson I+J human single fold scFv Library with minor modifications. A 1:1 mixture of propagated phage from DHF.a and DHF.c libraries (DHF a+c) was used for the selection in an ELISA microplate (Maxisorp, ThermoFisher Scientific) coated with 10 μg/mL of the bait antigen (EDIII Mut N or WT). The phage library was sequentially subtracted with wells coated with BSA and an unrelated Fc-fusion protein prior to the selection with EDIII antigens to control for nonspecific or Fc-specific binders. After the second and third rounds of selection, monoclonal scFv-phages were randomly picked for screening with phage ELISA.

### Focus Reduction Neutralization Test (FRNT) and Antibody-Dependent Enhancement (ADE) Assay

Focus reduction neutralization test and antibody-dependent enhancement assay were conducted as previously described.^13^ Briefly, a serial dilution of antibody was mixed with virus and incubated for 1 h at 37 °C. The mixture was transferred to monolayered Vero cells and incubated for 2h at 37 °C followed by an overlay of 1.3% CMC in MEM medium. The cells were incubated for 2 days at 37 °C. After fixation, permeabilization, and staining using an anti-E antibody 4G2, a 1:1000 dilution of a rabbit antimouse Ig conjugated with HRP (P0260, Dako) was used to stain the cells. Foci were developed with the DAB substrate. The 50% focus reduction neutralization titer (FRNT-50) was determined from a graph plotted between % neutralization versus the antibody concentration using a nonlinear regression curve (sigmoidal dose response) function (GraphPad Prism).

For the ADE assay, a serial dilution of antibody was mixed with virus and incubated for 1h at 37 °C. The antibody-virus mixture was then mixed with U937 cells to a multiplicity of infection (MOI) of 0.5 and incubated for 4 days. Afterward, U937 culture supernatant was collected and titrated on Vero cells as described above. Fold enhancement was calculated from the infectious titer in antibody-treated samples over the infectious titer of samples without antibody treatment.

## Supporting Information Available

Structure-guided selection of the N-glycosylation sites for selective epitope shielding, analysis of EDIII antigen with sequon mutations, analysis of EDIII antigen with sequon mutations, analysis of EDIII antigen with sequon mutations to shield 2C8 epitope, source image of Figure 3 and 4, deglycosylation of the monoglycosylated EDIII antigens, ELISA screening of phage clones from round 2 Mut N-selection (R2N), immunoglobulin germline gene analysis of similar scFv-phages from Mut N-selection and WT-selection, binding analysis of distinct hit scFv-phage from Mut N and WT selections, additional monoclonal phage ELISA screening of Mut N-selection round 3 (R3N) selection, mapping of scFv-phage binding residues with capture ELISA, analyses of purified IgG1 antibodies, screening and characterization of phage clones from Mut WT-selection by ELISA (PDF)
