# Supplementary material for: An Engineered N-Glycosylated Dengue Envelope Protein Domain III Facilitates Epitope-Directed Selection of Potently Neutralizing and Minimally Enhancing Antibodies
Source: ACS Infect Dis. 2024 Jun 29;10(8):2690–704. doi: 10.1021/acsinfecdis.4c00058 (PMC11320570; doi:10.1021/acsinfecdis.4c00058)
Supplement: Supplementary file 1 — id4c00058_si_001.pdf [file id4c00058_si_001.pdf]

Supporting Information

An Engineered N-Glycosylated Dengue Envelope Protein Domain III Facilitates Epitope-Directed Selection of Potently Neutralizing and Minimally Enhancing Antibodies.

Napon Nilchan[1,2], Romchat Kraivong[1,2], Prasit Luangaram[1,2], Anunyaporn Phungsom[1,2], Mongkhonphan Tantiwatcharakunthon[1,2], Somchoke Traewachiwiphak[1,2], Tanapan Prommool[1,2], Nuntaya Punyadee[3,4], Panisadee Avirutnan[3,4], Thaneeya Duangchinda[1,2], Prida Malasit[1,3,4], Chunya Puttikhunt[1,2]**

[1]Molecular Biology of Dengue and Flaviviruses Research Team, Medical Molecular Biotechnology Research Group and [2]Medical Biotechnology Research Unit, National Center for Genetic Engineering and Biotechnology (BIOTEC), National Science and Technology Development Agency (NSTDA), Pathum Thani 12120, Thailand.

[3]Siriraj Center of Research Excellence in Dengue and Emerging Pathogens and [4] Division of Dengue Hemorrhagic Fever Research, Faculty of Medicine Siriraj Hospital, Mahidol University, Bangkok 10700, Thailand.

*Corresponding authors: napon.nil@biotec.or.th and chunyapk@biotec.or.th

TABLE OF CONTENTS

	Page
**Supplementary Figure S1**<br>Structure-guided selection of the N-glycosylation sites for selective epitope shielding.	S3
**Supplementary Figure S2**<br>Analysis of EDIII antigen with sequon mutations.	S4
**Supplementary Figure S3**<br>Analysis of EDIII antigen with sequon mutations to shield 2C8 epitope.	S5
**Supplementary Figure S4**<br>Source image of Figure 3 and 4.	S6
**Supplementary Figure S5**<br>Deglycosylation of the monoglycosylated EDIII antigens.	S6
**Supplementary Figure S6**<br>Size-exclusion chromatography (SEC) traces of purified EDIII antigens.	S7
**Supplementary Figure S7**<br>Non-denaturing deglycosylation of EDIII WT and Mut N antigens.	S8
**Supplementary Figure S8**<br>ELISA screening of phage clones from round 2 Mut N-selection (R2N).	S9
**Supplementary Figure S9**<br>An immunoglobulin germline gene analysis of similar scFv-phages from Mut N-selection and WT-selection.	S10
**Supplementary Figure S10**<br>Binding analyses of distinct hit scFv-phage from Mut N and WT selections.	S10
**Supplementary Figure S11**<br>Additional monoclonal phage ELISA screening of Mut N-selection round 3 (R3N) selection.	S11
**Supplementary Figure S12**<br>Mapping of scFv-phage binding residues with capture ELISA.	S11
**Supplementary Figure S13**<br>Analyses of purified IgG1 antibodies.	S12
**Supplementary Figure S14**<br>Screening and characterization of phage clones from Mut WT-selection by ELISA.	S13

Supplementary Figure S1.

A

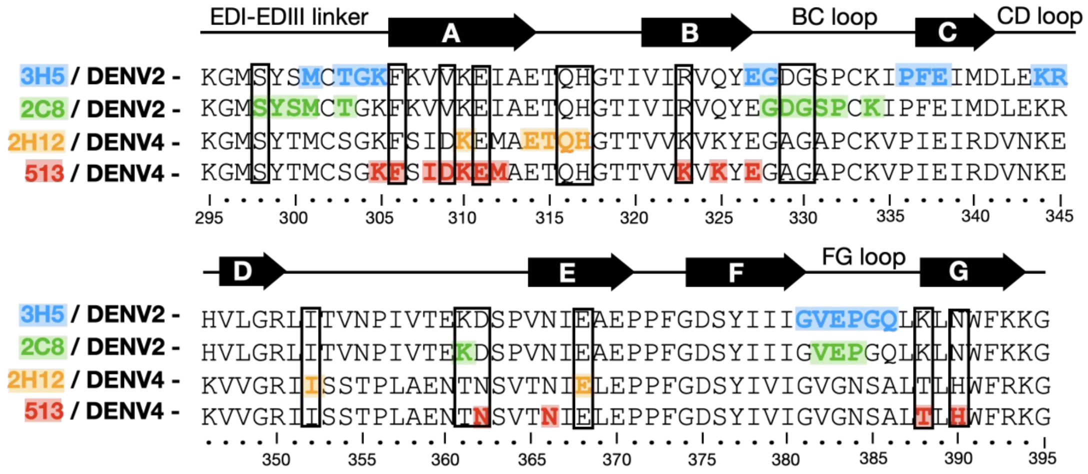

B

Antibody epitope	Epitope residue on DENV2 EDIII	Comments
2C8	S298	Selected as an N-glycosylation site
	D329	Selected as an N-glycosylation site
	G330	An introduction of sequon mutation (G330N_P332T) might disrupt the conformation of the BC loop (G is conformationally flexible, while P is rigid and is conserved in flaviviruses)
	K361	On the border of the interacting surface
2H12	E314	On the border of the interacting surface
	T315	On the border of the interacting surface
	Q316	Selected as an N-glycosylation site (on the A strand)
	H317	Selected as an N-glycosylation site (on the A strand)
	I352	Possible N-glycosylation site
	E368	Possible N-glycosylation site
513	F306	The side chain is inside the protein
	V309	Selected as an N-glycosylation site (on the AB loop)
	E311	Selected as an N-glycosylation site (on the AB loop)
	R323	Forms H-bonding with N366
	N362	possible N-glycosylation site, but 362 is part of the lateral ridge
	K388	Forms salt bridge with E383
	N390	On the border of the interacting surface
3H5	K305	Selected as an N-glycosylation site. 305 is a key residue for multiple lateral ridge targeting antibodies with potent neutralization activity[1]

Supplementary Figure S1. Structure-guided selection of the N-glycosylation sites for selective epitope shielding. (A) Epitope residues of four anti-EDIII template antibodies are highlighted in colors corresponding to each antibody. Residues that allow sequon NxS/T mutation without disrupting binding residues of other antibodies were displayed in boxes. (B) Analysis of the residues shown in boxes upon mapping onto a model of EDIII-antibody crystal structures. The selected residues for mutation are highlighted in colors.

Note: For 2H12 antibody, only 316 and 317 were initially picked because the residues locate right on the protruding AB loop. However, the two possible N-glycosylation sites at residue 352 and 368 were later cloned and shown to be glycosylated. While EDIII mutant 352N retained binding to 3H5, the mutant 368N showed dramatic binding reduction to 3H5. Both mutants were not further investigated.

Supplementary Figure S2.

A

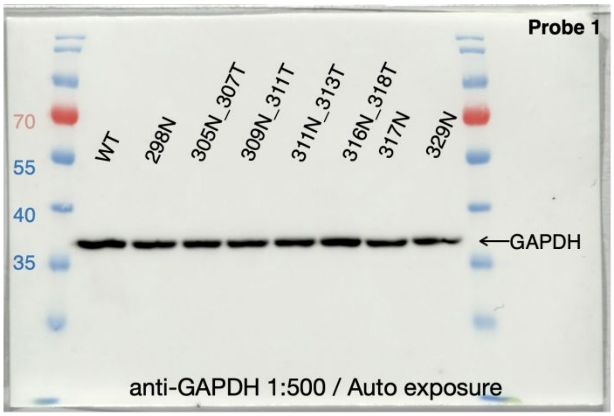

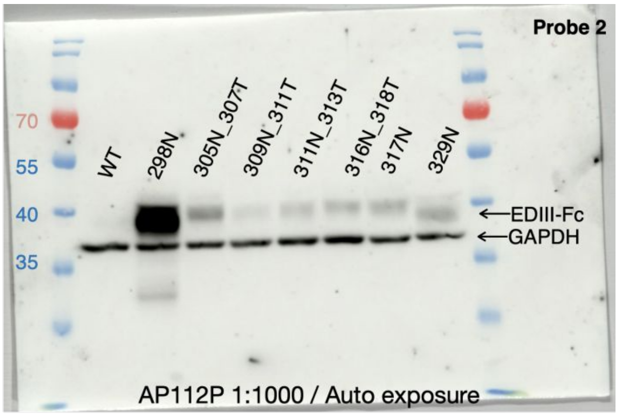

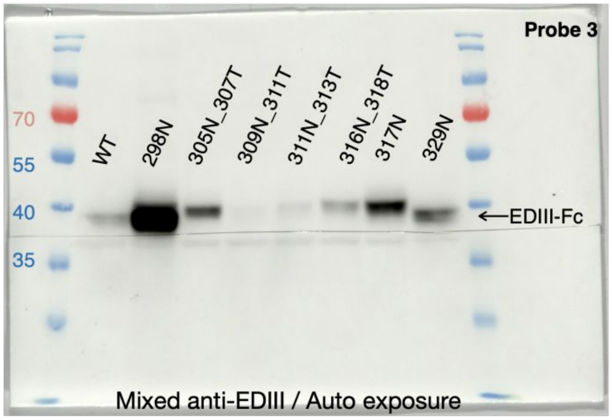

Probe	Primary antibody	Secondary antibody
1	mouse anti-GAPDH 1:500<br>4°C, overnight	anti-mouse Ig - HRP (1:2000)<br>r.t., 1h
2	goat anti-human IgG (H+L) - HRP<br>AP112P 1:1000<br>4°C, overnight	-
3	Mixed anti-EDIII<br>3H5 (hybridoma sup.)<br>2H12 (hybridoma sup.)<br>2C8 (hybridoma sup.)<br>513 (transfected 293T sup.)<br>r.t., 2h	anti-mouse Ig - HRP (1:2000)<br>+<br>anti-human kappa light chain - HRP<br>(1:4000)<br>r.t., 1h

Notes

The nitrocellulose membrane was not stripped between each probe.

sup. = neat supernatant from either hybridoma culture or transfected 293.

B

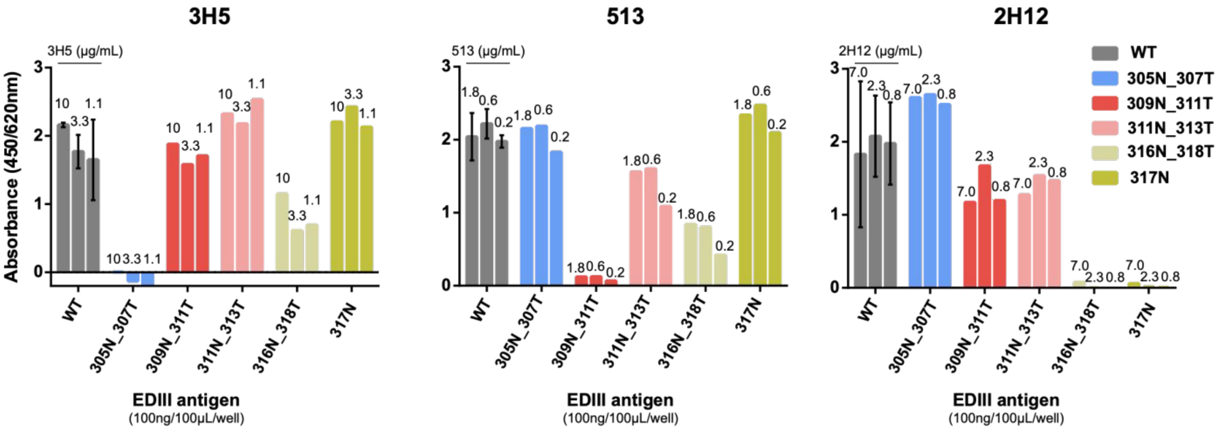

Supplementary Figure S2. Analysis of EDIII antigen with sequon mutations. (A) Western blot of transfected 293T cell lysate to confirm expression of glycosylated EDIII antigens. (B) Initial ELISA of purified glycosylated EDIII antigens with anti-EDIII antibody panel. Data are from a single experiment with two technical replicates only for EDIII WT (on two different ELISA plates). The error bars representing SD of the replicates.

Supplementary Figure S3.

A

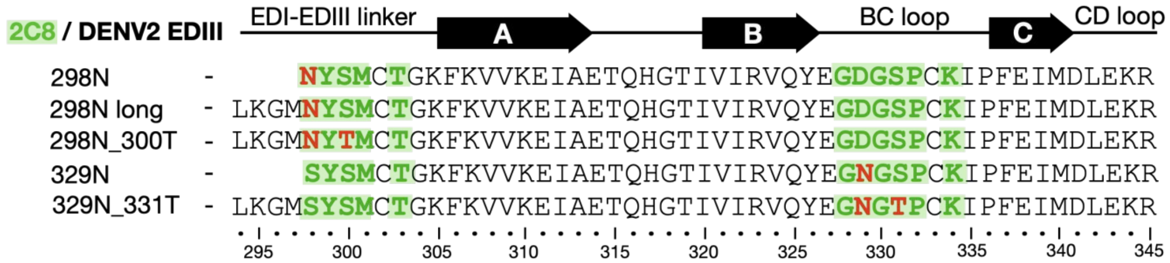

B

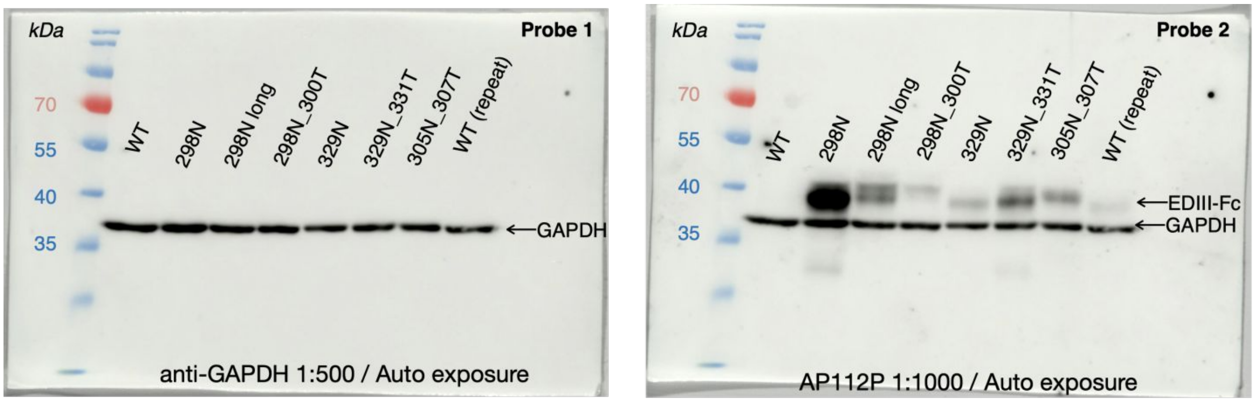

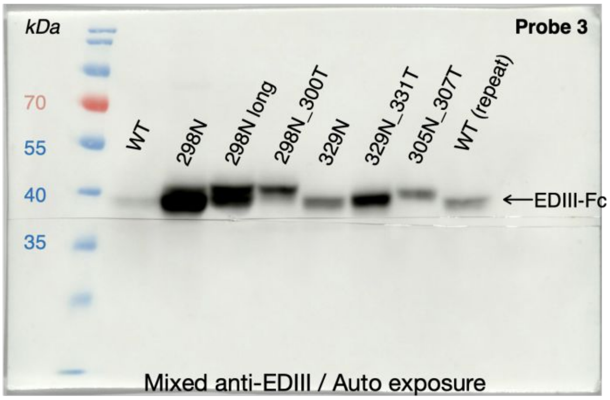

Probe	Primary antibody	Secondary antibody
1	mouse anti-GAPDH 1:500<br>4°C, overnight	anti-mouse Ig - HRP (1:2000)<br>r.t., 1h
2	goat anti-human IgG (H+L)<br>AP112P 1:1000<br>4°C, overnight	-
3	Mixed anti-EDIII<br>3H5 (hybridoma sup.)<br>2H12 (hybridoma sup.)<br>2C8 (hybridoma sup.)<br>513 (transfected 293T sup.)<br>r.t., 2h	anti-mouse Ig - HRP (1:2000)<br>+<br>anti-human kappa light chain - HRP<br>(1:4000)<br>r.t., 1h

Notes

The nitrocellulose membrane was not stripped between each probe.

sup. = neat supernatant from either hybridoma culture or transfected 293.

Supplementary Figure S3. Analysis of EDIII antigen with sequon mutations to shield 2C8 epitope. (A) A scheme comparing mutations on different EDIII antigen constructs to shield 2C8 epitope. (B) Western blot of transfected 293T cell lysate to confirm expression and glycosylation of EDIII antigens designed to shield 2C8 epitope. A monoglycosylated EDIII 305N_307T was used as a positive control.

Supplementary Figure S4.

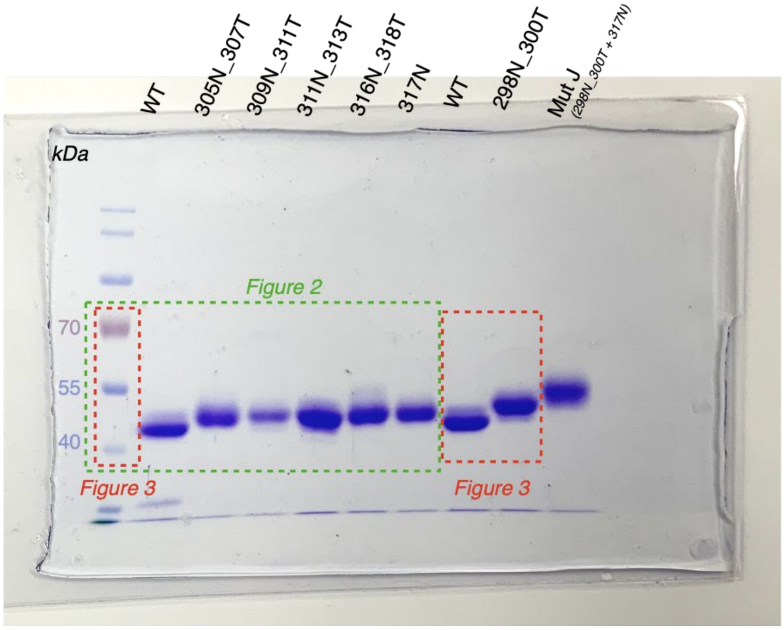

Supplementary Figure S4. Source image of Figure 3 and 4. SDS-PAGE analysis of purified EDIII-Fc antigens. Samples were heated with a 4x reducing loading buffer and heated at 95°C for 10min prior to the electrophoresis separation.

Supplementary Figure S5.

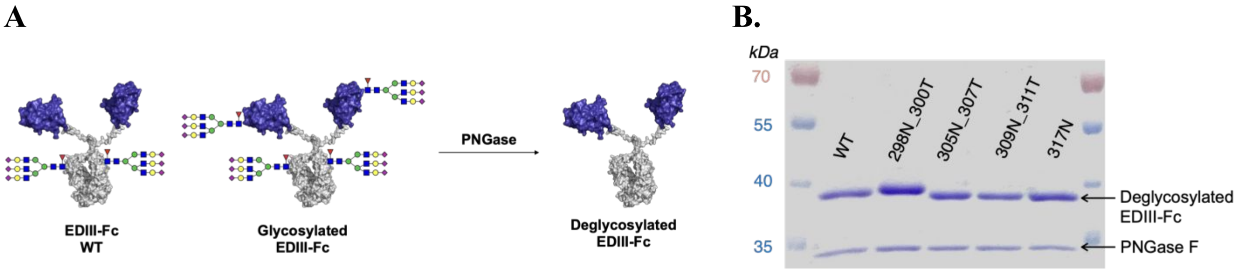

Supplementary Figure S5. Deglycosylation of the monoglycosylated EDIII antigens. (A) A reaction scheme of deglycosylation reaction by PNGase F enzyme on EDIII antigens. (B) SDS-PAGE analysis of deglycosylated EDIII antigen with PNGase F under denaturing reaction conditions and subsequently stained with Coomassie blue. We noted the slight difference in the apparent size of the deglycosylated mutant 298N_300T could possibly stem from an incomplete deglycosylation reaction or from a different amino acid length of the EDII antigen. While other EDIII antigens in the panel contain amino acid 298-394 of EDIII, the mutant 298N_300T contains amino acid 294-394 of EDIII. The additional 4 amino acids (^{294}LKGM^{297}), particularly Lys(K)-295 might affect the protein mobility of SDS-PAGE.

Supplementary Figure S6.

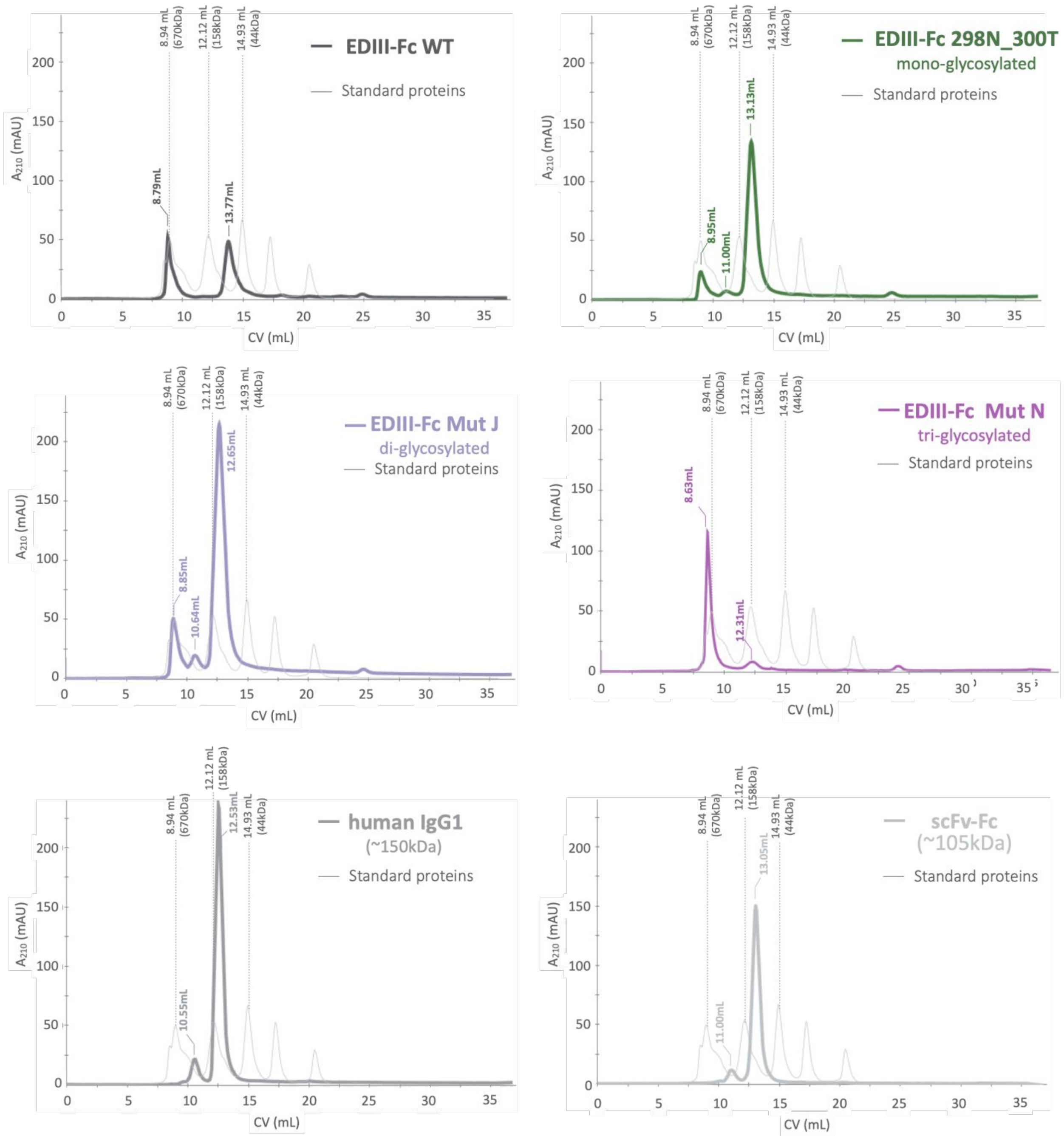

EDIII-Fc / Samples	% Integration larger MW peaks *(CV)*	% Integration lower MW peak *(CV)*
**WT**	**42%** *(8.79 mL)*	**58%** *(13.77mL)*
**298N_300T** (monoglycosylated)	**20%** *(8.95, 11.00mL)*	**80%** *(13.13mL)*
**Mut J** (di-glycosylated)	**19%** *(8.85, 10.64mL)*	**81%** *(12.65mL)*
**Mut N** (tri-glycosylated)	**95%** *(8.63mL)*	**5%** (12.31mL)
**scFv-Fc**	**7%** *(11.00mL)*	**93%** *(13.05mL)*
**human IgG1**	**8%** *(10.55mL)*	**92%** *(12.53mL)*

Supplementary Figure S6. Size-exclusion chromatography (SEC) traces of purified EDIII antigens. EDIII-Fc antigens with a calculated molecular weight (MW) of ~78kDa (Fc dimerization) are expected to elute between 12.12-14.93 column volume (CV, mL). The expected elution window was defined by peaks of standard protein mixture (158kDa bovine gamma-globulin and 44kDa ovalbumin). Lower MW peak was defined as protein eluted within the expected CV of 12.12 to 14.93 mL. Protein peaks that eluted earlier than 12.12 mL are expected to be an oligomeric state or soluble aggregates with higher MW. A human IgG1 antibody and an scFv-Fc antibody were used as control samples.

Supplementary Figure S7.

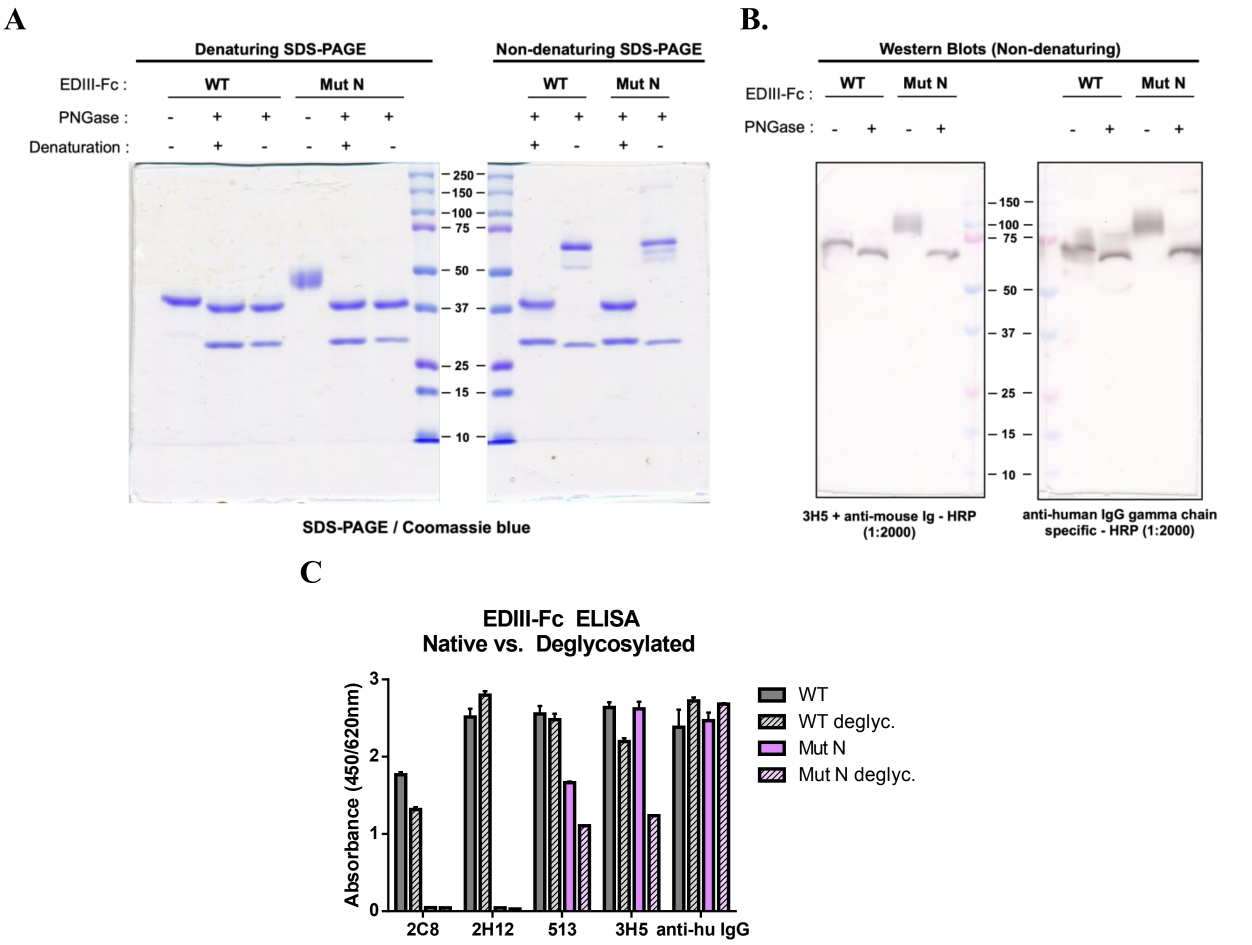

Supplementary Figure S7. Non-denaturing deglycosylation of EDIII WT and Mut N antigens. (A) SDS-PAGE analysis of deglycosylated EDIII antigen (+PNGase) under both denatured and non-denatured conditions to confirm complete deglycosylation. (B) Western blots of the deglycosylated EDIII antigens under non-denatured reaction conditions. (C) ELISA of the template anti-EDIII antibodies against EDIII WT and Mut N, with and without deglycosylation treatment (deglycosylated and native, respectively). An anti-human IgG antibody was used as a control to ensure comparable amount of the EDIII antigens in the assay. Data are from a single experiment. Error bars represent SD of two technical replicates.

Supplementary Figure S8.

R2N scFv-phage	EDIII antigens: Mut N	EDIII antigens: WT	Negative controls: Unrelated Fc fusion protein	Negative controls: BSA
**R2N_1G11**	2.64	2.41	0.05	0.07
**R2N_1A7**	0.34	0.05	0.05	0.06
R2N_1A1	0.64	0.09	0.09	0.07
R2N_1C1	0.48	0.09	0.07	0.06
R2N_1D1	0.52	0.08	0.06	0.07
R2N_1C2	0.69	0.09	0.09	0.07
R2N_1E2	0.79	0.12	0.14	0.08
R2N_1G2	0.62	0.09	0.10	0.08
R2N_1H2	0.65	0.16	0.10	0.06
R2N_1A3	0.51	0.07	0.06	0.06
R2N_1B5	0.80	0.09	0.11	0.07
R2N_1D5	0.40	0.09	0.08	0.07
R2N_1B8	0.23	0.06	0.05	0.06
R2N_1D8	0.24	0.06	0.05	0.06
R2N_1B11	0.19	0.07	0.05	0.06
R2N_1B1	0.73	0.08	0.08	0.06
R2N_1B2	1.07	0.13	0.09	0.07
R2N_1C3	0.44	0.08	0.07	0.06
R2N_1A4	0.35	0.06	0.06	0.06
R2N_1A5	0.59	0.08	0.07	0.06
R2N_1C5	0.69	0.10	0.09	0.07
R2N_1C7	0.58	0.10	0.08	0.10
R2N_1A8	0.75	0.07	0.06	0.08
R2N_1H8	0.54	0.08	0.06	0.06
R2N_1A12	0.38	0.09	0.06	0.07
neg ctrl	0.06	0.06	0.07	0.08

Incomplete scFv (either V_H or V_L): R2N_1A1 – R2N_1B11

Cannot read the seq.: R2N_1B1 – R2N_1A12

R2N_1G11, R2N_1A7: *contain complete scFv seq.*

Re-examine binding with a panel of EDIII antigens

Antigens	R2N_1A7: Rep1	R2N_1A7: Rep2	AP112P 1:4000
WT	0.06	0.08	3.47
298N_300T	0.06	0.07	3.72
305N_307T	0.08	0.08	3.48
309N_311T	0.06	0.06	2.95
317N	0.08	0.09	3.39
Mut N	0.10	0.10	3.37
unrelated Fc fusion	0.07	0.08	3.41
BSA	0.06	0.08	0.07

Supplementary Figure S8. ELISA screening of phage clones from round 2 Mut N-selection (R2N). Hits were initially defined as phage clones that show binding toward either EDIII Mut N or WT well above the negative controls of BSA and a Fc-fusion protein (Ranges from Abs = 0.05 – 0.14) We considered any phage that exhibited binding with Abs >0.14 to be reactive and the phagemids were sequenced. Only two phage clones, R2N_1G11 and R2N_1A7, contain complete scFv sequence. The other clones contain either V_H or V_L gene, or cannot be sequenced are regarded as false-positive. Only clone R2N_1G11 was shown to be a true hit, while R2N_1A7 were confirmed to be a false-positive upon re-examining it binding with a panel of EDIII antigens.

Supplementary Figure S9.

Hit scFv	V_H				V_L		
	V	J	D	CDR3	V	J	CDR3
**R3N_2D3**	V3-23*04 F	J4*02 F	D6-6*01 F	CAKGRYASPSPPFDPW	V3-21*04	J2*01	CQVWDSGNDSLGGS
**R2WT_1A12**	V3-23*04 F	J4*02 F	D6-6*01 F	CAKGRYASPSPPFDPW	V3-21*04	J3*01	CQVWDSGSDHWVF
**R3WT_2G3**	V3-23*04 F	J4*02 F	D6-6*01 F	CAKGRYASPSPPFDPW	V3-21*04	J5*01	CQVWDSGSVNL

Supplementary Figure S9. An immunoglobulin germline gene analysis of similar scFv-phages from Mut N-selection and WT-selection.

Supplementary Figure S10.

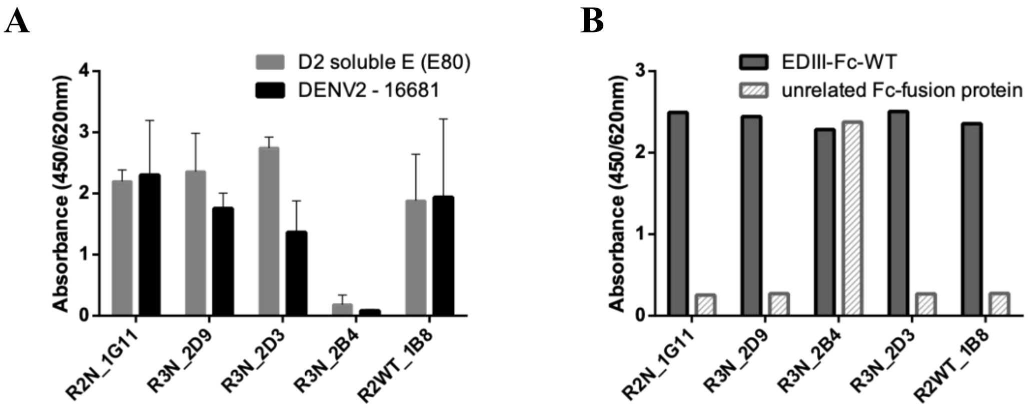

Supplementary Figure S10. Binding analyses of distinct hit scFv-phage from Mut N and WT selections. (A) Capture ELISA of hit scFv-phage with different DENV-2 antigens, soluble E protein (E80) and DENV virion. Data are from a single experiment with error bars represent SD of two replicates. (B) Capture ELISA EDIII mutants with scFv-phage results. Data shown are from a single experiment.

Supplementary Figure S11.

	NAME	WT	Mut N	Neg ctrl
identical scFv sequence to R2N_1G11	R3N_3F1	0.34	0.17	0.06
	R3N_3E3	1.42	0.21	0.06
	R3N_3G12	0.21	0.18	0.06
	R3N_3O2	0.52	0.17	0.06
	R3N_3J9	0.32	0.19	0.07
	R3N_3P10	0.23	2.87	0.07
	R3N_3O12	0.33	0.22	0.08
	R3N_3F11	0.18	0.12	0.08
identical scFv sequence to R3N_2B4	R3N_3E12	0.18	0.14	0.06

Supplementary Figure S11. Additional monoclonal phage ELISA screening of Mut N-selection round 3 (R3N) selection. All positive clones were sequenced and shown to contain identical scFv to either R2N_1G11 and R3N_2B4.

Supplementary Figure S12.

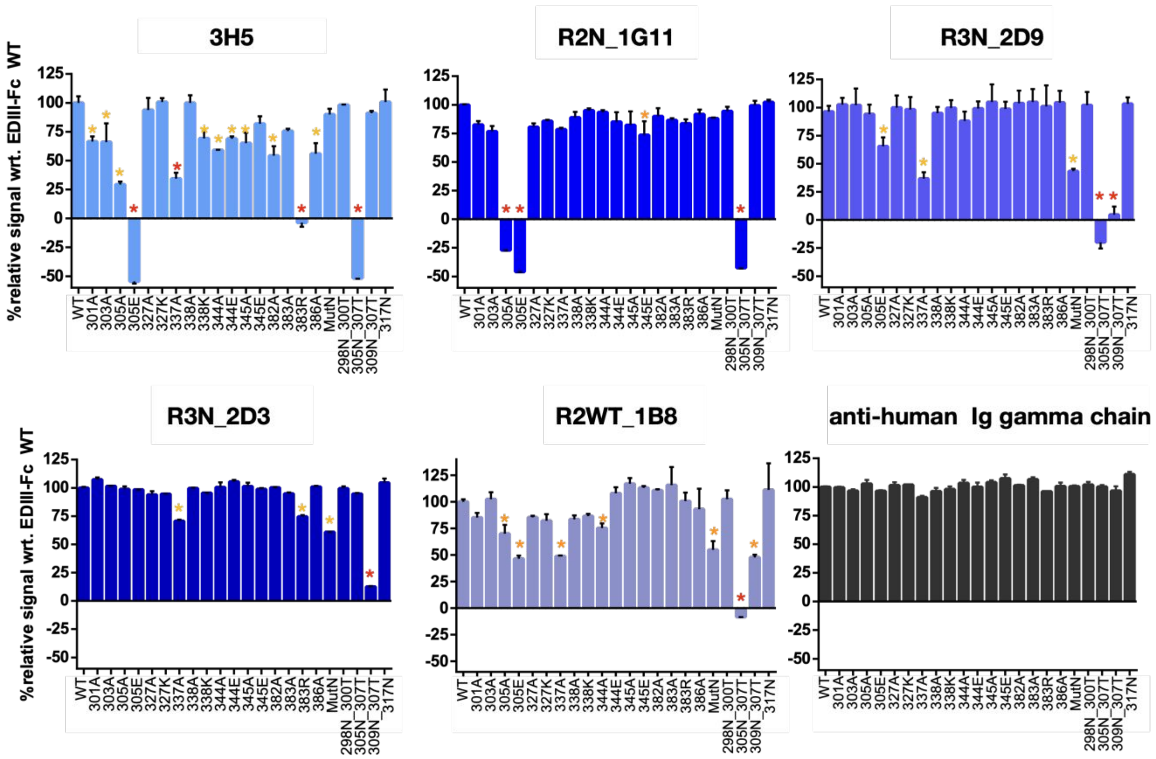

Supplementary Figure S12. Mapping of scFv-phage binding residues with capture ELISA. A panel of EDIII antigen individually mutated at 3H5 epitope residues and a panel of glycosylated EDIII antigens were used for the mapping. EDIII mutants with a relative signal lower than 25% (severe binding reduction) are marked with red asterisks, while those with relative signal between 75%-25% (moderate binding reduction) are marked with yellow asterisks. 3H5 antibody was used as a control experiment to assure that the assay was able to detect binding reduction resulted from the mutations. Anti-human Ig gamma chain antibody was used to ensure antigen loading across the mutant panel. Data are from a single experiment. Error bars represent SD of two technical replicates.

Supplementary Figure S13.

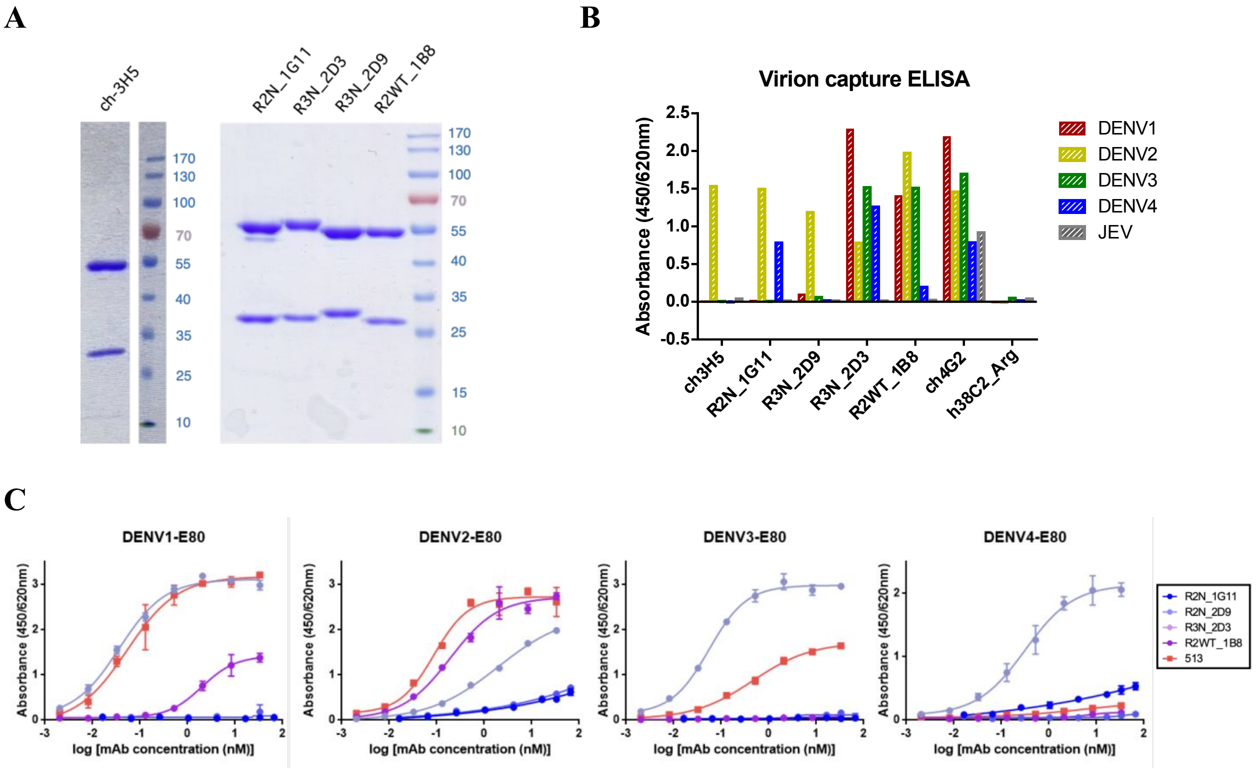

Supplementary Figure S13. Analyses of purified IgG1 antibodies. (A) An SDS-PAGE analysis of purified scFv-phage derived and chimeric antibodies under denaturing conditions and subsequently stained with Coomassie blue. (B) Capture ELISA of the purified antibodies with virion of DENV and Japanese encephalitis virus (JEV). Chimeric 4G2 (IgG1, anti-E flavivirus) and a humanized catalytic antibody h38C2_Arg were used as positive and negative controls, respectively. The representing data are from a single experiment. (C) Indirect ELISA with purified soluble envelope protein (E80) of DENV 1-4 with scFv-derived antibodies. A cross-reactive anti-EDIII 513 was used as a control antibody. The error bars represent SD from technical duplicates.

Supplementary Figure S14.

Initial monoclonal phage ELISA of distinct scFv-phage clones

NAME	WT	Mut N	Neg ctrl
R2WT_1C1	1.49	0.06	0.06
R2WT_1C3	2.52	0.06	0.05
R2WT_1C4	1.13	0.06	0.06
R2WT_1G9	2.98	0.07	0.05
R2WT_1G11	0.38	0.06	0.12
R3WT_2B2	1.36	0.06	0.05
R3WT_2C3	1.33	0.07	0.05
R3WT_2F3	1.75	0.051	0.051
R3WT_2F12	1.435	0.059	0.06
R2WT_1E3	0.82	0.07	0.05
R3WT_2A1	2.079	0.083	0.045

Re-examine binding with a panel of EDIII antigens

Group	scFv-phage	EDIII-Fc antigens						unrelated Fc-fusion protein	BSA
		WT	298N_300T	305N_307T	309N_311T	317N	MutN		
Reactive to only EDIII WT (in the initial screening)	R2WT_1C1	1.45	1.43	1.47	0.06	1.50	0.06	0.07	0.07
	R2WT_1C3	1.43	1.43	1.38	0.06	1.47	0.07	0.07	0.07
	R2WT_1C4	1.61	1.56	1.57	0.06	1.60	0.07	0.07	0.07
	R2WT_1G9	1.53	1.53	1.54	0.04	1.62	0.05	0.05	0.05
	R2WT_1G11	0.95	0.87	0.46	0.05	1.03	0.06	0.06	0.06
	R3WT_2B2 N	1.58	1.52	1.45	0.05	1.62	0.06	0.05	0.05
	R3WT_2C3	1.63	1.61	1.60	0.05	1.64	0.05	0.05	0.05
	R3WT_2F3	1.36	1.37	1.33	0.06	1.41	0.07	0.07	0.10
	R3WT_2F12	1.34	1.28	1.30	0.05	1.40	0.20	0.05	0.07
	R2WT_1E3	1.26	1.27	0.97	0.23	1.33	1.20	0.07	0.06
	R3WT_2A1	1.55	1.54	1.58	0.32	1.65	1.34	0.05	0.05
Reactive to both EDIII WT and Mut N (hit clones)	R2WT_1A12	1.36	1.39	1.40	1.28	1.40	1.54	0.07	0.06
	R3WT_2G3	1.41	1.35	1.35	1.33	1.36	1.53	0.07	0.07
	R2WT_1B8	1.47	1.41	0.09	1.01	1.47	1.63	0.07	0.07
	R2N_1G11	1.30	1.26	0.06	1.32	1.45	1.41	0.07	0.07
	R3N_2D9	1.37	1.30	0.11	0.84	1.43	1.47	0.06	0.07
	R3N_2D3	1.33	1.32	1.30	1.22	1.39	1.47	0.07	0.07
	AP112P	1.39	1.40	1.41	1.07	1.46	1.46	1.56	0.08

Supplementary Figure S14. Screening and characterization of phage clones from Mut WT-selection by ELISA. Unique scFv-phage clones from a phage screening that showed specific binding to EDIII WT but not Mut N was picked to re-examine for their putative binding region using a panel EDIII antigens. The tables show absorbance values at 450nm. The absorbance of the initial phage ELISA screening was from a single experiment. The absorbance values of the re-examination of binding (right table) are average values of two technical replicates from a single experiment. AP112P is a goat-anti human IgG-HRP which was used as a control to ensure comparable amount of immobilized EDIII-Fc antigens in the assay.
